# Hyperactivated Wnt Signaling Induces Synthetic Lethal Interaction with Rb Inactivation by Elevating TORC1 Activities

**DOI:** 10.1371/journal.pgen.1004357

**Published:** 2014-05-08

**Authors:** Tianyi Zhang, Yang Liao, Fu-Ning Hsu, Robin Zhang, Jennifer S. Searle, Xun Pei, Xuan Li, Hyung Don Ryoo, Jun-Yuan Ji, Wei Du

**Affiliations:** 1 Ben May Department for Cancer Research, The University of Chicago, Chicago, Illinois, United States of America; 2 Department of Molecular and Cellular Medicine, College of Medicine, Texas A&M Health Science Center, College Station, Texas, United States of America; 3 Department of Cell Biology, New York University School of Medicine, New York, New York, United States of America; Harvard Medical School, Howard Hughes Medical Institute, United States of America

## Abstract

Inactivation of the Rb tumor suppressor can lead to increased cell proliferation or cell death depending on specific cellular context. Therefore, identification of the interacting pathways that modulate the effect of Rb loss will provide novel insights into the roles of Rb in cancer development and promote new therapeutic strategies. Here, we identify a novel synthetic lethal interaction between Rb inactivation and deregulated Wg/Wnt signaling through unbiased genetic screens. We show that a weak allele of *axin*, which deregulates Wg signaling and increases cell proliferation without obvious effects on cell fate specification, significantly alters metabolic gene expression, causes hypersensitivity to metabolic stress induced by fasting, and induces synergistic apoptosis with mutation of fly Rb ortholog, *rbf*. Furthermore, hyperactivation of Wg signaling by other components of the Wg pathway also induces synergistic apoptosis with *rbf*. We show that hyperactivated Wg signaling significantly increases TORC1 activity and induces excessive energy stress with *rbf* mutation. Inhibition of TORC1 activity significantly suppressed synergistic cell death induced by hyperactivated Wg signaling and *rbf* inactivation, which is correlated with decreased energy stress and decreased induction of apoptotic regulator expression. Finally the synthetic lethality between Rb and deregulated Wnt signaling is conserved in mammalian cells and that inactivation of Rb and APC induces synergistic cell death through a similar mechanism. These results suggest that elevated TORC1 activity and metabolic stress underpin the evolutionarily conserved synthetic lethal interaction between hyperactivated Wnt signaling and inactivated Rb tumor suppressor.

## Author Summary

Inactivation of Rb tumor suppressor is common in cancers. Therefore, identification of genes and pathways that are synthetic lethal with Rb will provide new insights into the role of Rb in cancer development and promote the development of novel therapeutic approaches. Here we identified a novel synthetic lethal interaction between Rb inactivation and hyperactivated Wnt signaling and showed that this synthetic lethal interaction is conserved in mammalian systems. We demonstrate that hyperactivated Wnt signaling activate TORC1 activity and induce excessive energy stress with inactivated Rb tumor suppressor, which underpins the evolutionarily conserved synthetic lethal interaction. This study provides novel insights into the interactions between the Rb, Wnt, and mTOR pathways in regulating cellular energy balance, cell growth, and survival.

## Introduction

The Retinoblastoma protein Rb is a tumor suppressor inactivated in a broad spectrum of cancers [1], [2]. Rb functions mainly through binding to the E2F family of transcription factors and regulating the expression of diverse cellular targets involved in cell cycle regulation, DNA replication and repair, apoptosis, metabolism, as well as differentiation. Consistent with this, loss of Rb can lead to increased cell proliferation or increased cell death, depending on specific cellular contexts. Therefore identification and characterization of the genes or signaling pathways that can modulate the consequences of Rb loss in cell proliferation or cell death will significantly advance our understanding of the role of Rb in cancer development, and may potentially help the development of novel approaches for therapeutic interventions [3].

The function of Rb and E2F proteins are highly conserved and much simpler in *Drosophila*. These features, in conjunction with a plethora of sophisticated genetic tools, make *Drosophila* an ideal model to identify genes that modulates the consequences of Rb loss [4], [5]. Forward genetic screens have identified several genes that show synergistic effects on apoptosis or differentiation with *rbf* (fly Rb) mutation [6], [7], [8], [9], [10]. Of particular interest is the synthetic lethal interactions between *rbf* and *TSC* genes [10], [11], which is conserved in mammalian systems [10], [12]. TSC2 functions in a complex with TSC1 to inhibit TORC1 activity by promoting Rheb in the inactive GDP-bound form [13], [14]. Mutations of *TSC* induce hyperactive TORC1 activity, which leads to excessive cellular stress, including ROS and energetic stress, and causes synergistic cell death in conjunction with Rb inactivation [9], [10], [12]. Consistent with this, several recent studies demonstrate that Rb also plays important roles in cell metabolism and stress induction. In *Drosophila*, *rbf* mutation was shown to cause metabolic reprogramming and *rbf* mutants are sensitized to conditions that impose metabolic stress such as fasting, which can be rescued by glutamine supply [15]. In *C. elegans*, transcriptome analysis of wild type and Rb mutant under normal or starving conditions revealed that Rb is essential not only to repress stress-inducible and metabolic genes, but also to activate stress-resistant genes, mitochondrial genes, and potential insulin pathway antagonists [16]. Furthermore, studies using mouse embryonic fibroblasts (MEFs) from triple knock-outs of all three Rb family members show that Rb/E2F directly regulate genes involved in glutamine metabolism [17]. Taken together, these studies suggest that Rb has conserved functions modulating cellular metabolism as well as the sensitivity of cells to additional metabolic stresses induced by specific environmental or genetic conditions.

In the current study, we identify a novel synthetic lethal interaction between deregulated *Wg* signaling and *rbf* mutation through genetic screens in *Drosophila*. We show that mutation of *axin* (*axn*), a negative regulator of the *Wg* signaling, significantly alters the expression of metabolic genes and is hypersensitive to metabolic stress induced by fasting, which can be rescued by glutamine supply. We further demonstrate that deregulated *Wg* signaling increased TORC1 activity, which induced excessive metabolic stress and synergistic cell death with *rbf* mutation. Finally we show that inactivation of APC and Rb induces synergistic apoptosis in human cancer cells through a similar mechanism. These results provide an alternative explanation for the long standing but confusing observation that colorectal cancers, which have deregulated Wnt signals, generally preserve Rb function and may even have amplification of the Rb loci.

## Results

### A weak allele of *axn* induces synergistic apoptosis with *rbf* mutation without affecting photoreceptor differentiation in *Drosophila* eye discs

**Figure 1:**
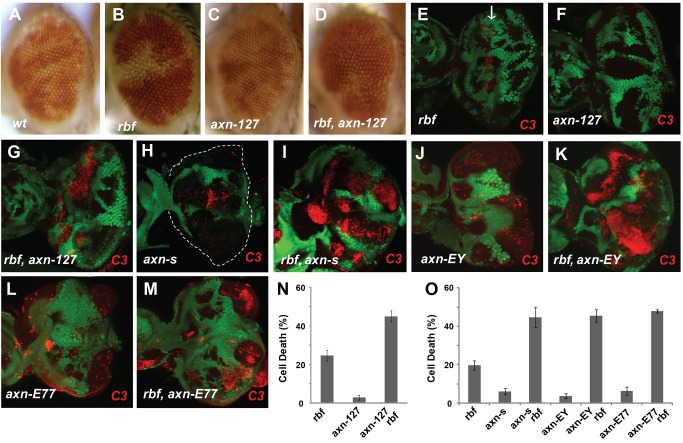
Synergistic cell death induced by *rbf* and *axn* mutations. Mosaic clones for mutations of the indicated genotypes in adult eyes are marked by white color, while wild type tissues are with orange color. Comparing to wild type control clones (A), *rbf* mutant clones were a bit smaller (B), while *axn^127^* mutant clones were similar to or even moderately larger (C). *rbf axn^127^* double mutant clones were mostly eliminated in adult eyes (D). In developing eye imaginal discs, mosaic clones are marked by the absence of GFP. Activated caspase-3 (C3) staining was used to detect apoptosis (or cell death). *rbf* mutation induced apoptosis just anterior to morphogenetic furrow (MF) indicated by white arrow (E). *axn^127^* mutation did not induce significant apoptosis (F), while *rbf axn^127^* mutations induced synergistic apoptosis in a broad region anterior to MF (G). Strong *axn* mutant alleles, *axn^S044230^* (*axn^S^*), *axn^EY10228^* (*axn^EY^*), and *axn^E77^*, induced low level apoptosis (H, J, L), and induced very strong apoptosis together with *rbf* mutation (I, K, M). (N) Quantification of C3 levels within *rbf*, *axn^127^*, and *rbf axn^127^* mutant clones anterior to the MF. (O) Quantification of C3 levels within indicated mutant clones in the whole eye discs. Error bars indicate standard deviations. Unless indicated otherwise, “synergistic apoptosis” means apoptosis induced by double mutations was significantly higher than apoptosis induced by either of the single mutant (P<0.0001).

In a genetic screen to identify mutations that can modulate *rbf* mutant phenotypes, we identified an EMS mutant *127*. In *Drosophila* adult eyes with mosaic clones, mutant clones are in white color and wild type cells in red color (Fig. 1A). Comparing to wild-type control clones, *rbf* single mutant clones were generally a bit smaller while *127* single mutant clones were similar to or moderately larger than WT clones (Fig. 1B–C). However, *rbf* and *127* double mutant clones were very small or undetectable in the adult eyes (Fig. 1D), suggesting that *rbf* and *127* mutations have synergistic effects against clonal growth or survival.

We tested whether the decreased amount of *rbf* and *127* (*rbf 127*) double mutant clones in adult eyes correlated with increased apoptosis in larval eye discs. Apoptosis in eye discs can be detected by the anti-cleaved caspase3 (C3) antibody. As shown previously [10], [18], [19], *rbf* mutation caused increased apoptosis just anterior to the morphogenetic furrow (MF) while little apoptosis was detected in wild type cells (GFP positive) at this stage (Fig. 1E). Although *127* mutant clones showed little apoptosis (Fig. 1F), *rbf* and *127* double clones located anterior to the MF exhibited significantly increased level of apoptosis compared to the single mutant clones (Fig. 1G, the results were quantified in 1N).

The *127* mutation was mapped to the *Drosophila* genomic region between 99D1-99E1 where the *axn* gene is located. Several evidences demonstrate that *127* mutation is an allele of *axn*: 1) *127* mutation failed to complement with the previously generated *axn* alleles; 2) DNA sequencing and mRNA RACE of the *axn* gene in *127* mutants revealed that Exon 10 of *axn* is linked to a repetitive heterochromatin sequence instead of Exon 11. Therefore the *axn* gene in 127 mutants encodes a protein lacking part of the DIX domain at the C-terminus (Supplementary Fig. S1A–D); 3) *127* homozygous mutants die at the pupal stage. Expression of wild-type *Axn* protein by *hs-Gal4/UAS-Axn* can partially rescue the pupal lethality, resulting in the development of adult flies without obvious defects; and 4) *127* mutant significantly increased Armadillo (Arm, fly β-catenin) protein levels (Supplementary Fig. S1E). Therefore, we renamed *127* as *axn^127^*. Since the phenotypes of *axn^127^* in lethality and in cell fate changes are much weaker than the previously reported *axn* alleles (see below), we consider *axn^127^* as a weak *axn* mutant allele.

To determine whether the *axn* mutation mediates the observed synergistic apoptosis phenotype with *rbf*, we tested the effects of the previously reported strong *axn* alleles, including *axn^EY10228^* (*axn^EY^*), *axn^E77^*, and *axn^S044230^* (*axn^S^*) [20], [21], [22]. Low level of apoptosis was observed in single mutant clones of these strong *axn* alleles, and much stronger apoptosis was observed in *axn*, *rbf* double mutant clones (Fig. 1H–M, results were quantified in 1O). Consistent with the notion that *axn^127^* is a weak allele, apoptosis in *rbf*- *axn^EY^*, *axn^E77^*, or *axn^S^* double mutant clones were observed in both anterior as well as posterior of the eye discs, while apoptosis in *rbf axn^127^* mutant cells were restricted to the region anterior to the MF.

The different patterns of apoptosis in eye discs are likely due to the different effects of the strong and the weak *axn* alleles on cell fate determination. Photoreceptor differentiation in eye disc can be detected by staining with the neuronal marker Elav. While the strong *axn* alleles blocked photoreceptor differentiation (Supplementary Fig. S2D) [21], [23], *axn* ^127^ did not (Supplementary Fig. S2B). In addition, *rbf* mutation did not have obvious effects on photoreceptor differentiation either alone or together with the *axn* alleles (Supplementary Fig. S2 A, C, E). To further compare the effects of the *axn* alleles on differentiation, we examined the effect of *axn* mutation on Senseless (Sens) expression, which is expressed in the SOPs along the presumptive wing margin [24]. We found that the strong *axn^EY^* mutation caused ectopic expression of Sens in wing discs, while *axn^127^* mutation did not (Supplementary Fig. S2 G, I). Again *rbf* mutation did not affect Sens expression either alone or together with the *axn* alleles (Supplementary Fig. S2 F–J).

Taken together, these data show that *axn^127^* does not affect photoreceptor differentiation in contrast to the previously identified strong *axn* alleles, and that *rbf* mutation has synergistic effects with *axn* on apoptosis but not on cell fate determinations.

### Deregulation of Wg signaling activity induces synergistic apoptosis with *rbf* mutation

**Figure 2:**
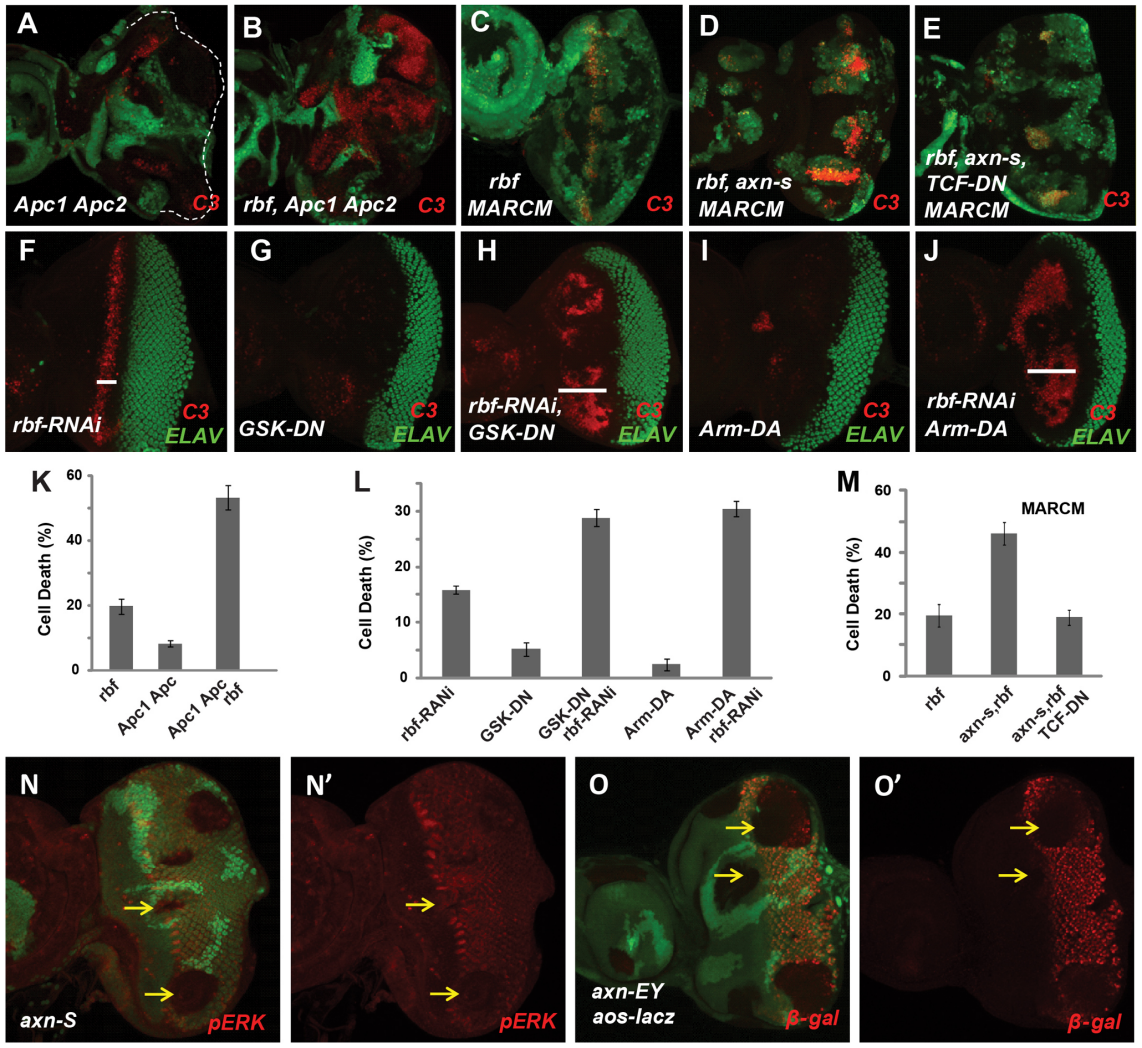
Hyperactivated *Wg* signaling induces synergistic apoptosis with *rbf* mutation. *Apc1 Apc2* mutations induced synergistic apoptosis with *rbf* mutation (A–B). Quantification of C3 levels within *rbf*, *Apc1 Apc2*, and *rbf Apc1 Apc2* mutant clones was shown in (K). Apoptotic pattern of *rbf* MARCM clones marked by the presence of GFP (C) was identical to regular mosaic clones marked by absence of GFP (Fig. 1E). MARCM clones that with *rbf axn^S^* mutations had increased apoptosis (D), which was inhibited by overexpressing of dominant negative TCF (TCF-DN) (E). Quantification of C3 levels within *rbf*, *rbf axn^S^*, and *rbf axn^S^ TCF-DN* MARCM clones was shown in panel (M). (F–J) Induction of dominant negative GSK3 (*GSK-DN*) or dominant active Armadillo (*Arm-DA*) expression with *rbf-RNAi* in most of the cells in eye discs starting at early L3 stage with heat shock FLP-out system. Depletion of *rbf* by RNAi induces apoptosis just anterior to MF (F). *GSK-DN* or *Arm-DA* expression alone did not induce significant level of apoptosis (G and I), but induced synergistic apoptosis with *rbf-RNAi* anterior to MF (H and J). White bars in (F, H and J) indicate that the apoptosis of *GSK-DN rbf-RNAi* or *Arm-DA rbf-RNAi* extended much more anterior than *rbf-RNAi* itself. Quantification of C3 levels anterior to MF in (F–J) is shown in panel (L). dp-ERK level in MF and posterior to MF are downregulated in *axn^S^* clones (N, N′). *aos-lacz* expression posterior to MF is downregulated in *axn^EY^* clones (O, O′).

To determine whether deregulated Wg signaling mediates the synergistic cell death effect of *axn* with *rbf*, we examined the effect of inactivating *APC* genes, which encode proteins that are in a complex with Axin protein to regulate β-catenin degradation and Wg signaling activity. As shown in Fig. 2, *Drosophila APC1-APC2* mutations also induce strong synergistic apoptosis with *rbf* mutation in eye discs (Fig. 2A–B, quantified in 2K). Therefore deregulation of the Wg signaling by inactivation of APC also induces synergistic apoptosis with *rbf* mutation.

We further tested the effect of deregulating Wg signaling by using dominant negative GSK3 (GSK-DN) or dominant active Armadillo (Arm-DA). Specifically, heat shock FLP-out approach was used to express GSK-DN or Arm-DA with or without *rbf*-RNAi in the whole eye discs at early L3 larval stage when photoreceptor differentiation has initiated in the posterior eye disc (Fig. 2F–J, samples with GFP shown in Fig. S1F). With this approach, *rbf*-RNAi induced a stripe of apoptosis just anterior to MF (Fig. 2F), while expression of GSK-DN or Arm-DA alone did not induce obvious apoptosis (Fig. 2G, 2I). However, GSK-DN or Arm-DA together with *rbf*-RNAi induced apoptosis in a broad region anterior to MF (Fig. 2H, 2J, quantified in 2L), which is similar to the apoptosis induced by *axn^127^ rbf* mutations. Therefore, deregulation of Wg signaling using dominant-negative GSK3 or dominant-active Armadillo also induce synergistic cell death in conjunction with *rbf* inactivation. Furthermore, inhibiting Wg signaling by expressing dominant negative TCF (TCF-DN) significantly inhibited synergistic cell death observed in *axn* ^S^ *rbf* double mutant clones (Fig. 2C–E, 2M), indicating that synergistic cell death of *axn* and *rbf* double mutants depends, at least in part, on the transcriptional activities of Arm/TCF.

Synergistic apoptosis was also observed in *axn rbf* mutant clones in wing discs (Supplementary Fig. S2K–N), although the apoptotic levels were significantly lower than those observed in eye discs (Fig. 1K, 1O). This difference is likely associated with the different effects of *axn* mutation on differentiation in the wing and eye discs. As discussed above, the strong *axn* mutations promote wing margin SOP cell fate as shown by ectopic *Sens* expression (Fig. S2I–J) while suppress photoreceptor differentiation in eye discs as shown by the block of Elav expression (Fig. S2D–E). The EGFR pathway is an important survival signal that is coupled with photoreceptor and SOP differentiation [25], [26], [27], [28]. We found that EGFR signaling activities, reflected by pERK antibody staining and *Argos-lacZ* reporter expression, are downregulated in and posterior to the MF in eye discs (Fig. 2N–O′) but are upregulated in wing discs in *axn* mutant clones (Supplementary Fig. S2O–P′). Therefore the differential effects of *axn* mutation on EGFR signaling in eye and wing tissues likely influenced the level of *axn rbf* synergistic cell death.

In summary, these results show that hyperactivation of the Wg signaling in conjunction with *rbf* mutation induce synergistic apoptosis in developing imaginal discs, and that the level of apoptosis is also influenced by tissue-specific effects of Wg signaling on cell differentiation and survival signaling.

### *axn rbf* synergistic cell death depends on upregulated Rheb/TORC1 signaling activities

We determined the effect of *axn* mutation on cell growth by comparing the ratio of individual mutant clone area over the corresponding WT twin spot area. Although *axn^127^* and other strong *axn* mutations have different effects on cell fate determination, all *axn* mutant clones show increased clone growth compared to WT controls (Supplementary Fig. S3 A–D).

**Figure 3:**
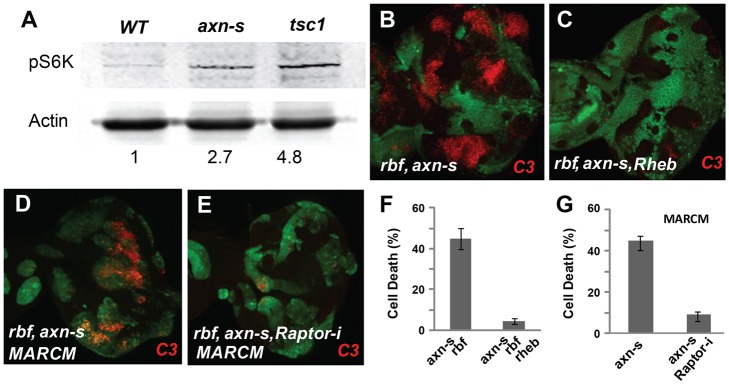
*axn rbf* synergistic cell death is mediated by deregulation of mTOR activities. pS6K signal was used to detect mTOR activities. *FRT 82B WT* control, *axn^S^* and *tsc1* mutant eye discs were generated by inducing clones in *Minute* background. pS6K in *axn^S^* mutant eye discs was increased and similar to that in *tsc1* mutant eye discs (A). *Rheb* mutation blocked apoptosis in *rbf axn^S^* mutant cells (B, C). Knockdown of Raptor by RNAi (*Raptor-i*) rescued *rbf axn^S^* cell death (D–E). Quantification of C3 levels in (B–C and D–E) was shown in panel (F) and (G) respectively.

One important growth and proliferation regulator in fly imaginal discs is the TSC-Rheb-TOR pathway. To determine whether TOR signaling is affected by *axn* mutation, we examined the phosphorylation of S6K, a direct target of TORC1, in eye discs that consist of mostly *axn* or *tsc1* mutant clones. The level of phospho S6K was significantly increased in *axn* mutant eye discs and similar to that of the *tsc1* mutant discs, which was used as a positive control (Fig. 3A). Therefore TORC1 signaling activity is significantly increased by mutation of *axn*.

A previous study showed that deregulated TORC1 increased dE2F1 protein level and promote S phase entry [11]. Indeed, increased expression of PCNA-GFP, an E2F reporter, was observed in both the strong and the weak *axn* mutant clones (Supplementary Fig. S3 E–F′). In addition, increased dE2F1 protein and increased BrdU incorporation were also observed in *axn* mutant clones (Supplementary Fig. S3G–H′).

Since previous studies showed that high TORC1 activities induced synergistic apoptosis with *rbf* mutation [10], [11], we tested whether increased TORC1 signaling activity contributes to synergistic cell death in *axn rbf* double mutant cells. Inhibition of TORC1 activity by mutation of Rheb, a direct upstream activator of TORC1, significantly decreased apoptosis in *axn* ^S^ *rbf* mutant cells (Fig. 3B–C, quantified in 3F). Similarly, knockdown of Raptor, a component of TORC1 complex, also significantly suppressed apoptosis in *axn* ^S^ *rbf* double mutant clones (Fig. 3D–E, quantified in G).

These results suggest that inactivation of *axn* leads to increased TORC1 signaling activity, which contributes to synergistic cell death in conjunction with *rbf* mutation.

### *axn rbf* mutant cells are energy deficient, and loss of LKB1 enhances apoptosis of *axn* or *axn rbf* mutant cells

**Figure 4:**
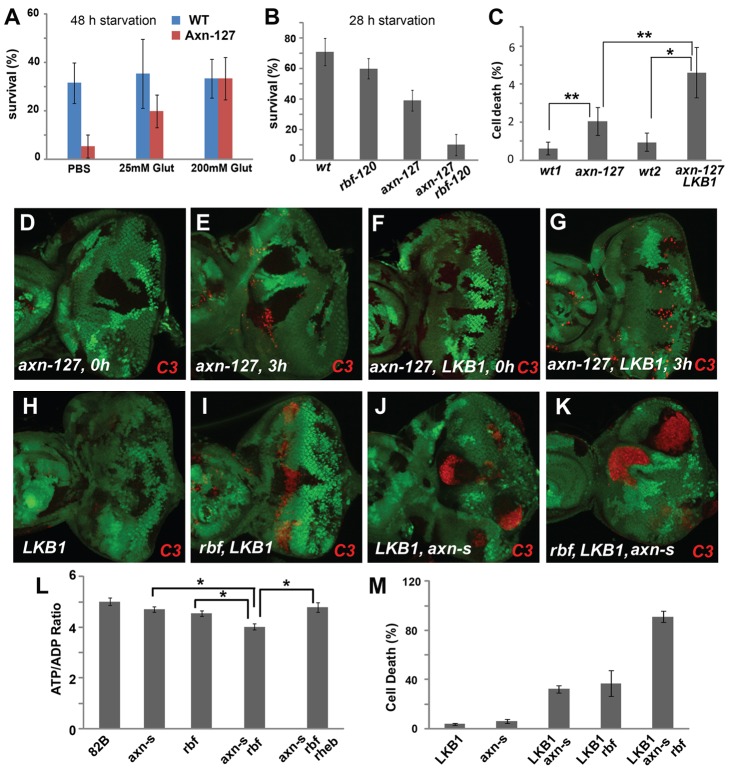
*axn* mutants are hypersensitive to fasting and energy deficiency. (A) Compared to the *FRT 82* controls, fewer *axn^127^* mutant larvae survived after starvation in PBS for 48 hours, and addition of glutamine to PBS largely rescued the lethality of the *axn^127^* mutant larvae (p = 0.0002 for PBS vs 25 mM Gln and p<0.0001 for PBS vs 100 mM Gln). (B) With 28 h starvation, survival rates of *rbf^120^* larvae or *axn^127^* larvae were lower than *WT* larvae, and *rbf^120^ axn^127^* double mutant larvae were much more sensitive to fasting than single mutant larvae (P<0.001 for *axn^127^* VS *rbf^120^ axn^127^*). (C) Quantification of cell death in eye discs fasted for 3 hours. In developing eye discs with mosaic mutant clones, discs without fasting showed very low level of apoptosis in both wild type tissues and *axn^127^* or *lkb1 axn^127^* mutant clones (D and F). Discs fasted for 3 hours had increased apoptosis in both wild type tissues and mutant mosaic clones (E and G), which are quantified in (C). The level of apoptosis in *axn^127^* or *lkb1 axn^127^* clones with fasting was significantly higher than surrounding wild type cells (p = 0.0015 for WT vs *axn^127^* and p<0.0001 for WT vs *lkb1 axn^127^*), and *lkb1 axn^127^* mutant cells have more apoptosis than *axn^127^* mutant cells (P<0.002) (C). *lkb1* single mutant did not show significant level of apoptosis (H), *lkb1 rbf* or *lkb1 axn^S^* mutations induced synergistic apoptosis (I–J), and *lkb1 axn^S^ rbf* triple mutant cells had very high levels of apoptosis (K). Quantification of C3 levels in (H–K) is shown in panel (M). The ATP/ADP ratio of eye discs with *axn^S^*, *axn^S^ rbf*, or *axn^S^ rbf rheb* mutant clones induced in *Minute* background were determined (L). Comparing to wild type cells (*FRT 82B*), *axn^S^* mutant cells had slightly lower ATP/ADP ratio (p<0.01); the ATP/ADP ratio of *axn^S^ rbf* mutant cells was significantly lower than that of the *axn^S^* or *rbf* mutant cells (p<0.0001); the ATP/ADP ratio of *axn^S^ rbf rheb* mutant cells was similar to that of the *axn^S^* mutants (p = 0.4, between *axn^S^* and *axn^S^ rbf rheb*) (L). * and ** indicate p<0.0001 and p<0.002, respectively.

Deregulated activation of TORC1 by Tsc1 or Tsc2 mutation causes an imbalance between the metabolic demand and supply, and the Tsc1/Tsc2 mutant cells are highly dependent on glutamine metabolism for survival during energy stress [29]. Similarly, *rbf* mutants were found to exhibit altered glutathione metabolism and are hypersensitive to energy stress induced by fasting [15]. The observed effect of *axn* mutant on TORC1 signaling prompted us to test whether *axn^127^* mutant larva also show hypersensitivity to fasting. Interestingly, *axn^127^* mutant larva are much more sensitive to fasting than the controls (*FRT 82B*) and addition of glutamine to PBS largely suppressed the observed sensitivity of the *axn^127^* mutants (Fig. 4A). The increased sensitivity of *axn^127^* mutant to fasting correlated with increased sensitivity to fasting-induced cell death in *axn^127^* mutant clones, which is enhanced by *lkb1* mutation (Fig. 4C–G). LKB1 is a kinase that functions to balance cellular energetic needs and supply through AMPK-dependent and-independent pathways. Inactivation of LKB1 has been shown to increase death of cells under energy stress [9]. Taken together, these observations suggest that *axn^127^* mutants have an altered metabolic process and show increased sensitivity to energy stress induced by fasting. Consistent with this notion, genome-wide expression studies using third instar *axn^127^* homozygous mutants showed that the top functions affected in *axn^127^* mutant include genes involved in metabolism, oxidation-reduction, stress response, signal transduction, and developmental processes (Supplemental Tables S1, S2). We generate *axn^127^ rbf^120^* double mutants to further test whether *rbf axn* mutations show synergistic effects in hypersensitivity to fasting. *rbf^120^* is a viable weak *rbf* allele [30]. Consistent with previous reports [15], more *rbf^120^* larvae died than WT control after 28 hour fasting. *axn^127^* larvae were also more sensitive than WT control to fasting. Interestingly, *rbf^120^ axn^127^* double larvae were even more sensitive to fasting than either *axn^127^* or *rbf^120^* single mutants (Fig. 4B).

To test if excessive metabolic and energy stress contribute to the synergistic cell death of *axn rbf* double mutants similar to that observed for the *rbf tsc2* mutant cells, we first determined whether *axn* single and *axn rbf* double mutant cells were under energy stress. The ATP/ADP ratio of eye discs with *axn*, *axn rbf*, or *axn rbf Rheb* mutant clones in *Minute* background were determined. Compared to wild-type cells (*FRT 82B*), *axn* mutant cells had slightly lower ATP/ADP ratio (P<0.01), suggesting that they were under mild energy stress. The ATP/ADP ratio of *axn rbf* mutant cells was significantly lower than that of the *axn* mutant cells (P<0.0001), indicating that the double mutant cells were under severe energy stress (Fig. 4L). Interestingly, blocking TORC1 activation by Rheb mutation increased the ATP/ADP ratio of *axn rbf* mutant cells to a level similar to that of the *axn* mutants (Fig. 4L, Fig. S4, p = 0.4, between *axn* and *axn rbf rheb*), suggesting that inhibition of TORC1 activity decreased energy stress of the *axn*,*rbf* mutants.

We further tested whether *lkb1* mutation showed synergistic effects with *axn* or *axn rbf*. Although *lkb1* single mutant did not show significant levels of apoptosis, *lkb1* mutation induced synergistic cell death with *axn^S^* mutation and *lkb1 axn rbf* triple mutant cells had very high levels of cell death (Fig. 4G–J, quantified in 4M).

Taken together, these results suggest that *axn* mutants are under energy stress and require the LKB1 pathway for survival. In addition, it is likely that excessive metabolic stress of *axn*,*rbf* mutants contributes to the synergistic cell death.

### *axn* and *rbf* mutations synergistically upregulate *Hid* expression, which is blocked by inhibiting TORC1 activity

**Figure 5:**
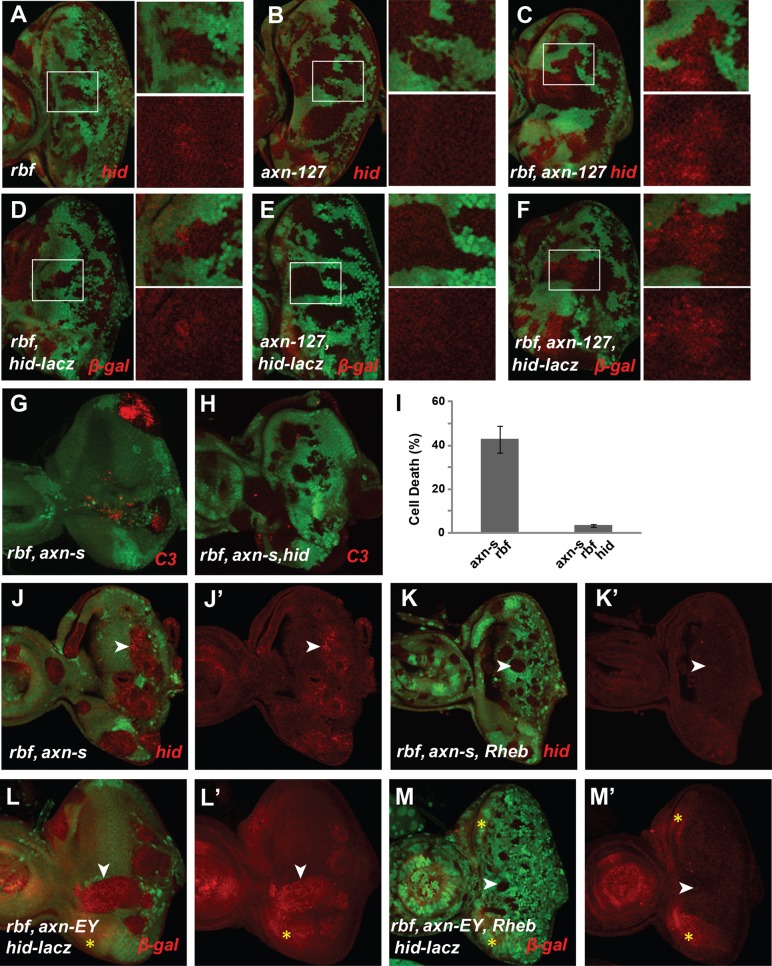
*axn* and *rbf* mutations synergistically upregulate *Hid* expressing depending on deregulated TORC1 activity. *rbf* mutation upregulated *Hid* protein and *hid-lacz* expression just anterior to MF (A and D), *axn^127^* did not change *Hid* protein or *hid-lacz* expression (B and E), while *rbf axn^127^* double mutations induced *Hid* protein or *hid-lacz* expression in a broad region anterior to MF region (C and F). *hid* mutation rescued *rbf axn^S^* apoptosis (G–H). Quantification of C3 levels was shown in panel (I). *rheb* mutation blocked upregulation of *Hid* protein in *rbf axn^S^* mutant clones (J–K′) and *Hid-lacZ* expression in *rbf axn^EY^* mutant clones (L–M′). White arrowheads in J–M′ point to different levels of Hid (J–K′) or β-gal (L–M′) in *rbf axn^S^* and *rbf axn^S^ rbeb* mutant clones. The yellow stars in L–M′ mark the background red channel signals at the lateral sides of the discs.

Hid is a critical regulator of apoptosis in *Drosophila* imaginal discs, and is induced by diverse developmental and stress signals including cell competition and DNA damage [31], [32]. Rbf-E2f1 directly regulates *Hid* expression [8], [33]. However the upregulated *Hid* expression and Hid protein level in *rbf* mutant clones were relatively weak and limited to the stripe just anterior to MF where *rbf* apoptosis occurs (Fig. 5A, 5D). Mutation of *axn^127^* alone did not affect *Hid* transcription or Hid protein levels (Fig. 5B, 5E). Interestingly, significantly expanded *Hid* transcription and Hid protein were observed in *axn^127^ rbf* double mutant clones anterior to the MF (Fig. 5C, 5F), which correlated with the observed synergistic apoptosis of these cells (Fig. 1G and N). We further tested the effect of strong *axn* alleles on Hid. Both *Hid* expression and Hid protein were significantly upregulated in *axn* ^S^ as well as in *axn* ^S^ *rbf* double mutant clones, however it is difficult to tell if the Hid expression is synergistically upregulated in the double mutant clones (Fig. 5J, L and data not shown).

To determine if Hid induction contributes to the synergistic cell death observed in *axn*,*rbf* double mutant clones, *axn*,*rbf* mutant clones were induced in the *hid* mutant background. As shown in Fig. 5G–I, mutation of *hid* largely blocked apoptosis of the *axn^S^,rbf* double mutant cells, demonstrating the critical role of Hid induction to synergistic cell death of *axn^S^,rbf* mutant cells.

Since blocking TORC1 activity blocks synergistic apoptosis of *axn*,*rbf* mutants, we tested the effect of inhibiting TORC1 on Hid induction. We observed that inhibiting TORC1 signaling by a *rheb* mutation strongly blocked induction of *Hid* transcription as well as accumulation of Hid protein (Fig. 5J–M′, white arrowheads), suggesting that induction of Hid in *axn*,*rbf* mutant clones is TORC1 dependent. Since TORC1 activity significantly alters cellular metabolic and energetic demand and supply and inhibition of TORC1 helps to restore the energy balance in *axn*,*rbf* mutant cells (Fig. 4L), these results suggest that Hid induction and apoptosis in *axn*,*rbf* mutant cells is regulated, at least in part, by metabolic and energy stress, similar to the synergistic cell death of *tsc2,rbf* mutant cells.

### Inactivation of APC and Rb synergistically induce cell death in mammalian cells

**Figure 6:**
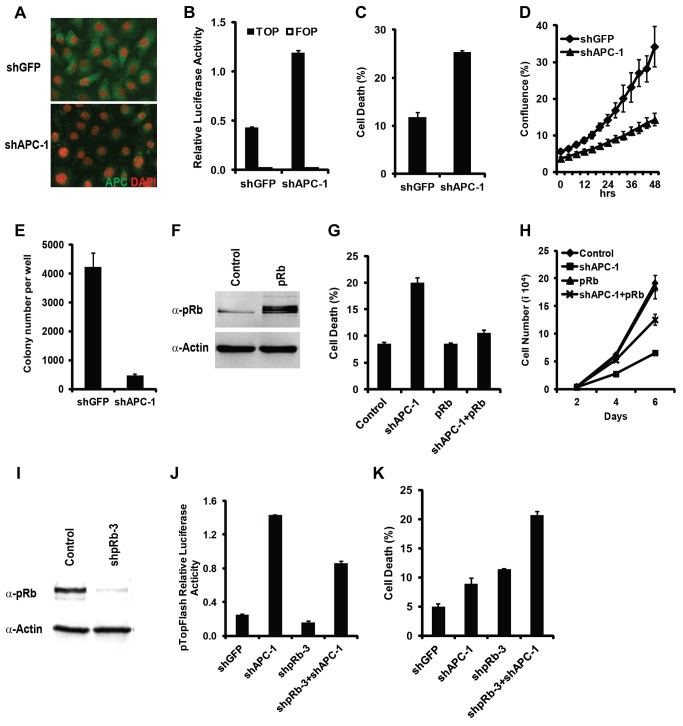
Hyperactivation of Wnt signaling and inactivation of Rb induced synergistic cell death effect in mammalian cells. In Rb mutant Du145 cells, knockdown of APC strongly reduced the level of APC protein detected by antibody staining (A) and increased the Wnt signaling reporter activity detected with the TOP-FOP luciferase assay (B). Knockdown of APC enhanced cell death (C), decreased the viable cell numbers (D), and inhibited the colony growth in soft agar (E). Overexpression of Rb restored WT pRb in Du145 cells (F), decreased the APC knockdown-induced cell death (G), and partially rescued the cell proliferation defect (H). (I–K) In HCT116 cells, knockdown Rb using shRb-3 significantly reduced the endogenous pRb level (I). (J and K) The effect of knockdown Rb and APC on Wnt pathway activity detected with the TOP-FOP luciferase assay (J) and on cell death detected by Annexin V staining (K).

The Rb/E2F and the Wnt signaling pathways are highly conserved between fly and mammalian systems. To determine whether deregulated Wnt signaling and Rb inactivation can also induce synergistic cell death in mammalian cells, we first determined whether activation of Wnt signaling can induce cell death in DU145 cells, a Rb mutant prostate cancer cell line [34]. Knockdown of Wnt signaling negative regulator APC using shRNA constructs strongly reduced the level of APC protein as shown by antibody staining (Fig. 6A) and increased the Wnt signaling reporter activities (Fig. 6B, Supplementary Fig. S5A). To determine whether deregulation of Wnt signaling by APC knockdown induced cell death, we stained cells with an early apoptosis marker Annexin V together with the nucleic acid dye propidium iodide. We observed that depletion of APC significantly increased cell death in DU145 cells (Fig. 6C, Supplementary Fig. S5B). In addition, knockdown of APC in DU145 cells significantly decreased viable cell numbers (Fig. 6D, Supplementary Fig. S5C), and decreased the colony growth in soft agar (Fig. 6E, Supplementary Fig. S5D). To determine whether the observed shAPC-induced death depends on the absence of Rb function, we investigated the effect of expressing WT Rb in APC knockdown DU145 cells. Expression of the transduced WT Rb can be easily detected (Fig. 6F). Expression of WT Rb significantly decreased APC knockdown-induced death (Fig. 6G), and partially restored the total viable cell numbers (Fig. 6H). Taken together, these results demonstrate that knockdown of APC significantly induced the cell death, which is dependent on the absence of Rb function.

Colorectal cancer cells commonly have deregulated Wnt signaling and intact Rb/E2F pathway [35]. Consistent with a previous report [36], knockdown of Rb in HCT116 colorectal cancer cells leads to decreased Wnt signaling reporter activity (Fig. 6I–J, Supplementary Fig. S5E–F) and increased cell death (Fig. 6K, Supplementary Fig. S5G). Rb knockdown-induced cell death in colorectal cancer cells was attributed to the reduced Wnt signaling activity [36]. To determine whether Rb knockdown induced cell death in HCT116 cells was due to reduced Wnt signaling or due to synergistic cell death induced by deregulated Wnt signaling and Rb inactivation, we set to distinguish these two possibilities in cells with depleted APC. Knockdown of APC significantly increased the Wnt signaling in HCT116 cells (Fig. 6J, Supplementary Fig. S5F), indicating that APC significantly inhibited Wnt signaling even though these cells contain a β-catenin gain of function mutant allele. Importantly, Wnt signaling reporter activity was higher in APC and Rb double knockdown cells than that in control knockdown cells (Fig. 6J, Supplementary Fig. S5F). However, increased Wnt signaling in the double knockdown cells did not suppress Rb knockdown-induced cell death. In fact, the cell death in Rb and APC double knockdown cells was even higher than those of the single or control knockdown cells (Fig. 6k, Supplementary Fig. S5G). Therefore, although Rb depletion decreases Wnt signaling activity in colorectal cancer cells, its induction of cell death is likely mediated by the synergistic death effect from pRb inactivation and deregulated Wnt signaling.

### Synergistically cell death induced by deregulated Wnt signaling and Rb inactivation requires TORC1 activity and involves oxidative stress induction

**Figure 7:**
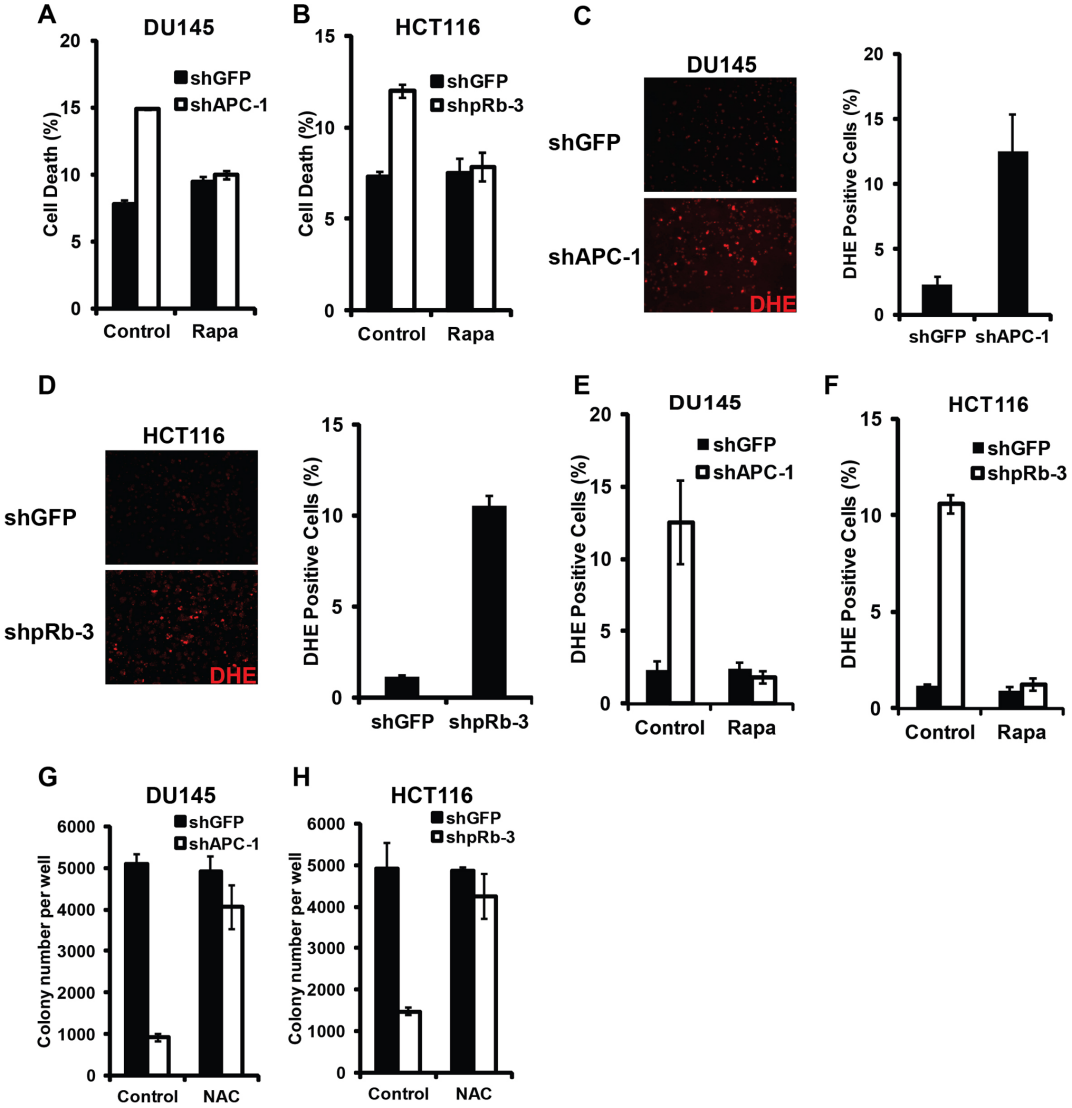
Synergistic cell death induced by hyperactivated Wnt signaling and Rb inactivation require TORC1 activity and involve ROS induction. Synergistic cell death from APC knockdown in DU145 cells (A) and Rb knockdown in HCT116 cells (B) was rescued by Rapamycin, an inhibitor of TORC1 activity. Both APC-knockdown DU145 cells (C) and Rb-knockdown HCT116 cells (D) grown in soft agar showed increased ROS level by DHE staining. (E–F) Rapamycin blocked ROS induction in APC-knockdown DU145 cells (E) and in Rb-knockdown HCT116 cells (F). (G–H) NAC, an antioxidant, rescued the ability of APC-knockdown DU145 (G) and Rb-knockdown HCT116 (H) cells to form colonies in soft agar.

Synergistic cell death from inactivated Rb and deregulated Wg signaling in *Drosophila* depends on upregulated TORC1 activity (Fig. 3). To determine whether TORC1 activity also contributes to the synergistic cell death in mammalian cells, we determined the effect of inhibiting mTORC1 activity using rapamycin. Rapamycin potently blocked APC knockdown induced cell death in Rb mutant DU145 cells as well as Rb knockdown induced cell death in HCT116 cells (Fig. 7A, B). These observations suggest that, similar to *Drosophila*, TORC1 activity is required for synergistic cell death induced by Rb inactivation in conjunction with deregulated Wnt signaling in mammalian cells.

Our previous studies have shown that inactivation of Rb and TSC2, a negative regulator of TORC1, induced synergistic cell death in cancer cells through induction of excessive cellular stress, including oxidative stress [10]. We used DHE, a dye that detects superoxide, to determine whether oxidative stress is also associated with deregulated Wnt signaling and Rb inactivation induced cell death. As shown in Fig. 7, highly elevated levels of DHE fluorescence were observed in APC-knockdown DU145 cells as well as in Rb-knockdown HCT116 cells grown in soft agar (Fig. 7C–D). Furthermore, Rapamycin, which inhibits TORC1 activity, suppressed the ROS level in these knockdown cells (Fig. 7E–F). Finally, NAC, a ROS scavenger, strongly rescued the knockdown-induced colony growth defects in soft agar (Fig. 7G–H). Taken together, these observations suggest that Rb inactivation and deregulated Wnt signaling induced cell death requires TORC1 activity and involves oxidative stress induction.

## Discussion

This study revealed a novel and evolutionarily conserved synthetic lethal interaction between hyperactivated Wnt signaling and inactivated Rb. We show that a weak allele of *axn* with deregulated Wg signaling significantly alters the expression of metabolic genes and is hypersensitive to metabolic stress induced by fasting in *Drosophila*. Furthermore, we observe that hyperactivation of Wg signaling significantly increased TORC1 activity and induced excessive energy stress and synergistic cell death in conjunction with *rbf* mutation. These observations are consistent with the previous studies, which showed increased TORC1 activity by *tsc1* or *tsc2* mutation induced synergistic apoptosis with Rb mutation [10], [11]. Our previous studies showed that mutation of *lkb1*, a key regulator of energy metabolism under energy stress conditions, promoted synergistic cell death with *rbf tsc1* mutations [9]. Similarly, we show here that *axn rbf* cells are also energy deficient and *lkb1* mutation strongly enhanced the apoptotic effects of *axn* or *rbf axn* mutants. Interestingly, inhibition of TORC1 activity significantly suppressed synergistic cell death induced by deregulated Wg signaling and *rbf* inactivation, which correlated with decreased energy stress and decreased induction of apoptotic regulator *Hid*. These results provide further evidence that excessive metabolic and energetic stress contributes to the synergistic cell death. Finally we demonstrate that the phenotypes and mechanisms of *axn rbf* synergistic apoptosis in *Drosophila* are conserved in mammalian cells and that inactivation of Rb and APC induces synergistic cell death that requires TORC1 activity and involves oxidative stress induction.

Wnt/Wg signaling is one of the key developmental signaling pathways repeatedly used in different developmental settings to regulate cell proliferation, apoptosis, as well as cell differentiation. The consequence of deregulated Wnt signaling depends on particular cellular contexts. In *Drosophila* larval eye discs, Wg signaling is essential for proliferation of the progenitor cells anterior to the MF. Mutant clones of *axn^127^*, which does not affect cell type specification or patterning, showed *Hid* upregulation and synergistic cell death with *rbf* only in the anterior proliferating region. In contrast, strong *axn* alleles, which blocks photoreceptor differentiation, caused synergistic cell death with *rbf* in both the anterior and the posterior clones. Therefore, it appears that synergistic cell death of deregulated Wnt signaling and *rbf* inactivation is mainly observed in the proliferating progenitor cells. Consistent with this, we found that the observed synergistic cell death is associated with increased TORC1 activity, metabolic stress, and cell proliferation.

In mammalian systems, Wnt signaling plays important roles in maintaining stem cell and progenitor cell homeostasis and deregulated Wnt signaling is observed in many types of cancers, particularly the colorectal cancers. It is quite likely that synergistic cell death interactions between deregulated Wnt signaling and inactivated Rb potentially play important roles in maintaining stem cell homeostasis as well as during cancer development. While Wnt signaling is required to maintain intestine stem cells, hyperactivation of Wnt signaling results in increased cell proliferation as well as increased apoptosis [37], [38]. Similarly inactivation of APC in hematopoietic stem cells (HSCs) increases cell proliferation as well as apoptosis, leading to HSC exhaustion and bone marrow failure [39]. Since pRb is inactivated during G1/S transition, pRb is partially inactivated as these stem cells or progenitor cells proliferate. An interesting possibility is that different levels of Wnt signaling activation or pRb inactivation will cause graded levels of metabolic alterations. When combined Wnt signaling hyperactivation and pRb inactivation induced metabolic change past a certain threshold, excessive metabolic stress and cell death will be induced. It is interesting to note that although Rb inactivation is found in almost half of cancer cells, colorectal cancers often show *Rb* copy gains with high level of Rb expression [35]. Since deregulated Wnt activities is the key cancer initiating event that exists in almost all colorectal cancer cells, the high Rb level can potentially prevent cell death induced by hyperactivated Wnt signaling, particularly during early cancer progression. In addition to inducing synergistic cell death with deregulated Wnt signaling, high E2F activities were also found to antagonize Wnt signaling by degrading β-catenin in a GSK3β independent manner [36]. It is possible that the Rb-E2F and Wnt signaling pathway may crosstalk at multiple levels, and Wnt signaling can induce either pro-apoptotic or survival signals depending on particular cellular context.

The observed synergistic cell death between hyperactive Wnt signaling and inactivated Rb may also contribute to the cancer cells drug sensitivity. A recent study showed that upregulation of Wnt signaling is required for cell death induction in melanoma cells by PLX4720, a selective inhibitor of activated BRAF(V600E). PLX4720 increased Wnt signaling and induced Bim expression and cell death in A375 melanoma cells, which was blocked by β-catenin (*CTNNB1*) siRNA [40]. A375 cells have lost the expression of p16INK4a, which is a cyclin dependent kinase (CDK) inhibitor that regulates the phosphorylation of pRb by D-type CDKs [41]. Therefore, pRb is likely at least partially inactivated in these cells. Interestingly, analysis of the Genomics of Drug Sensitivity in Cancer database [42], a publicly available IC50 dataset of 147 anticancer agents on over 1000 tumor cell lines, revealed that PLX4720 was one of the seven drugs that show increased effectiveness toward cancers that have genomic alterations of the Rb gene [43]. Therefore, it will be interesting to investigate whether Wnt induced apoptosis in A375 cells requires Rb inactivation.

Deregulated TORC1 activity is often observed in cancers and inhibition of TORC1 activity can potentially be used as a strategy to inhibit cancer growth. However, the clinical trials of the TORC1 inhibitor Rapamycin and its derivatives have only seen very limited success in small subset of cancers [44]. Besides the possibilities that these inhibitors are not potent enough to completely inhibit TORC1 or they activate feedback signaling, our studies raise the possibility that inhibition of TORC1 decreases the stress levels in cancer cells and promotes cancer cell survival. Indeed, decreasing the activities of TORC1 or its downstream target S6K partially rescues the Rb- TSC synergistic cell death [10], [11].

Several studies described how increased Wnt signaling activates TORC1 activity. One possible mechanism is mediated by the inhibition of mTOR by GSK3 through the phosphorylation of TSC2 [45], [46], [47], [48], [49]. In this case, increased Wnt signaling will activate mTOR by inhibiting GSK3. Another mechanism described recently is that GSK3 and mTOR cooperate to regulate S6K phosphorylation [50]. Additionally, canonical Wg signaling has been shown to promote insulin sensitivity by upregulating insulin receptor expression [51]. Therefore, Wnt and TOR signaling pathways intersect at multiple levels.

## Materials and Methods

### *Drosophila* stocks

Fly stocks used in this study include: *rbf^15aΔ^* [8], *dtsc1^29^* [52], *lkb1^X5^* [53], *hid^138^* [8]. *axn^EY10228^* (BL17649), *axn^E77^* [21], *axn^S044230^* [54], *APC1^Q8^ APC2^79^* [54], *Hid-lacz* [55], *Rheb^4L1^* (BL39737), *UAS-Axn-GFP* (BL7224), *UAS-Raptor RNAi* (BL34814), *UAS-Rbf RNAi* (BL36744), *UAS-Arm^S10^* (BL 4782), *aos-lacz* (BL2513), *UAS-TCF^DN^* (BL4785), *UAS-Ras^V12^* (BL4847), *UAS-GSK3DN, PCNA-GFP* [56].

### Genetic screen for mutations that modulate the phenotypes of *rbf* mutant

Ethyl methanesulfonate (EMS)-induced screen to identify mutations that can modulate the phenotypes of *rbf* was carried similar as described [8], except that w; *p{ry+, neoFRT82B}* males were used for mutagenesis, and *rbf^15aΔ,^w*, *eyFLP; p{ry+, neoFRT82B} p{w+, Ubi-GFP} p{w+, rbf-G3}* and *w*, *eyFLP; p{ry+, neoFRT82B} p{w+, Ubi-GFP}* stocks were used for screening and *rbf* dependence test.

### 3′ RACE

Total RNA was isolated with TRIzol (Invitrogen). cDNA was synthesized with 1 µg total RNA, M-MLV Reverse Transcriptase (Invitrogen), and 3′RACE-T7 primer (5′-TAATACGACTCA CTATAGGGTTTTTTTTTTTTTTTTTTTTTTTV-3′ (V = A, G, or C)). Nested PCR was first performed with the Axin3′middleF primer (5′-CGGGTGTGGAAGGACCAAA-3′) and T7 primer (5′-TAATACGACTCACTATAGGG-3′), and then the Axin3′middleF-2 primer (5′-ATTCCGGAATGGTCAGCGA-3′) and T7 primer. PCR products were gel purified and sequenced.

### Immunostaining

Immunostaining was performed at room temperature unless indicated otherwise. Larval imaginal discs were dissected in 1× PBS, fixed with 4% formaldehyde in PBS for 25 min, washed twice with 1× PBS with 0.3% Triton-X100 (PBST), and incubated with primary antibody in blocking solution (PBST plus 5% normal goat serum) overnight at 4°C. Primary antibodies used: rabbit anti-activated Caspase-3 (C3, 1∶300 from Cell Signaling), mouse anti-β-Galactosidase (1∶100, DSHB), rat anti-ELAV (1∶50, DSHB), Guinea pig anti-Senseless [57], and Guinea pig anti-E2F1 (Orr-Weaver lab). Guinea pig anti-Hid antibody was affinity purified with recombinant GST-Hid [58]. Following incubation with primary antibody, samples were washed three times (10 minutes each) in PBST, and incubated with secondary antibodies from Jackson ImmunoResearch (1∶200 to 1∶400). Sample was mounted in 70% Glycerol with 1,4-diazabicyclo[2.2.2]octane (DABCO) at 12.5 mg/mL. For mammalian cell staining, infected cells were seeded onto glass coverslips, and processed for staining. Fixed, permeabilized, and blocked cells were incubated with rabbit anti-APC M2 (kindly provided by Kristi Neufeld, University of Kansas), followed by FITC-coupled secondary antibody. Imaging was done with the Zeiss Axioscope/ApoTome microscope using the AxioCam CCD camera controlled by Zeiss Axiovision software. In experiments with internal controls (for example, the WT tissues from the same disc that do not show cell death), the exposure time for each sample were determined using the “measure” function in Axiovision for each channel to get optimal exposure without signal oversaturation. For experiments with no internal controls, exposure time was fixed using the genotype with brightest signal to avoid overexposure.

### Quantification of cell death levels in developing imaginal discs

Cell death (%) is determined as described previously [10] by the percentage of clone area (pixels) that have above background level of caspase 3 (C3) signal using the Histogram function in Photoshop. The background level of C3 signal was determined as the level that is equal or below 99% of the C3 signal in the WT tissues that have no apoptosis. The Average and standard deviation of percent cell death for each genotype discs was then determined and compared.

### Western blot

40 *Drosophila* eye discs with each specific genotype were dissected in insect cell media CCM3 (Hyclone), and moved to 1.5 ml tubes with 100 µl 1× SDS-PAGE loading buffer immediately. The samples were pipetted for several times, boiled for 5 minutes, quickly centrifuged, and 20 µl of them were loaded for SDS-PAGE. For mammalian samples, cells were washed twice with 1× PBS, and lysed in RIPA buffer (50 mm Tris.Cl [pH 7.4], 150 mm NaCl, 2 mm EDTA, 1% NP40, 0.1% SDS, 0.5% sodium deoxycholate, plus protease inhibitors). Primary antibodies are rabbit anti-pS6K (1∶300, Cell Signaling), mouse anti β-actin (1∶1000 Santa Cruz), and mouse anti-Rb 4.1 (1∶10, Developmental Studies Hybridoma Bank). The goat anti-mouse IgG and goat anti-rabbit IgG secondary antibodies were obtained from Li-Cor. Western detection was carried out using a Li-Cor Odyssey image reader.

### ATP∶ADP ratio determination

Eye imaginal discs with specific genotypes were dissected, pipetted with 120 uL 1× Passive Lysis Buffer (Promega) for 15 times in a 1.5 mL tube on ice, boiled for 5 minutes, then incubated on ice for 2 minutes. After centrifugation at 18,000G for 2 minutes, 20 uL of each sample was used to assay the ADP∶ATP ratio using the Enzylight kit according the manufacturer's protocol (BioAssay Systems).

### Whole larvae and imaginal discs starvation

To induce metabolic stress, *FRT 82B Axn^127^* and *FRT 82B* control 2nd instar larvae were collected at 72 hour after egg lay, rinsed to remove any residual fly food, and transferred into empty vials containing one 11 cm by 21 cm Kimwipe soaked with 1 ml of either 1× PBS or PBS with glutamine. Eight vials containing 25 larvae each were used per genotype per condition. These vials were incubated at 25°C for 48 hours, at which point the Kimwipe was extracted and the larvae were characterized. *Drosophila* larvae were determined to be viable if they responded to stimuli from poking with a blunted pair of forceps. For eye disc starvation, dissected eye discs were left in 1× PBS at room temperature for 3 hours before fixation, and eye discs fixed immediately after dissection were used as control (0 hr). The immunostaing with C3 antibody is the same as described above.

### Genome-wide gene expression analysis

Larvae of Oregon R *Drosophila* (control) and *axn^127^* homozygous mutants (*w^1118^; +; FRT82B axn^127^*) were collected at the third instar wandering stage. Total RNA was extracted from three larvae per sample with 1.0 ml of TRIzol Reagent (Life Technologies Corporation) according to the manufacturer's instructions. The microarray analysis was performed according to the protocol that was described previously [59]. The complete sets of microarray data have been deposited in the ArrayExpress database (http://www.ebi.ac.uk/arrayexpress/; accession number is E-MTAB-2342). Gene Ontology was performed with GO-TermFinder (http://amigo.geneontology.org/cgi-bin/amigo/term_enrichment) [60].

### Cell culture

DU145 and HCT116 cells were obtained from the American Type Culture Collection. All the cells were maintained in Dulbecco's modified Eagle's medium supplemented with 10% fetal bovine serum (FBS, Atlas Biologicals), 50 IU penicillin/streptomycin, and 2 mmol/l L-glutamine (Invitrogen) in a humidified atmosphere with 5% CO2 at 37°C.

### Plasmids and lentiviral preparation and transduction

Human pRb was subcloned into the lentiviral expression vector pCDHCMV-EF1-puro (System Biosciences). The pLKO.1 lentiviral RNAi expression system was used to construct lentiviral shRNA. The sequences of shRNA used in this study included the following:

shAPC-1: 5′-CCGGTGAGGTCATCTCAGAACAAGCTCGAGCTTGTTCTGAGATGACCTCtttttt-3′

shAPC-2: 5′-CCGGTAAGACGTTGCGAGAAGTTGGACTCGAGTCCAACTTCTCGCAACGTCTTtttttt-3′

shpRb-1: 5′-CCGGCGACGAGTCAAACAAGCCAATCTCGAGATTGGCTTGTTTGACTCGTCGTTTTTG

shRb-3: 5′-CCGGTGGTTGTGTCGAAATTGGATCACTCGAGTGATCCAATTTCGACACAACCTTTTTT-3′

shGFP: 5′-CCGGTACGTCTATATCATGGCCGACAACTAGTTGTCGGCCATGATATAGACGTTTTTTG-3′

The shGFP was used as a control in this study. Viral packaging was done according to the previously described protocol [10]. Briefly, expression plasmids pCMV-dR8.91 and pCMV-VSV-G were cotransfected into HEK293T cells using the calcium phosphate method at 10∶5∶5 µg (for a 10-cm dish). The transfection medium containing calcium phosphate and plasmid mixture was replaced with fresh complete medium after incubation for 6 hr. Media containing virus was collected 48 hr after transfection, and then concentrated at 19,400 g for 2 hr. The virus pellet was re-dissolved, and stocked at −80°C. Cells were infected with the viruses for 48 hr, and were treated as described.

### FACS analysis of cell death

Quantification of cell death was performed using FACSCanto (BD Biosciences) after cells were stained with Annexin V-FITC (BD Biosciences) and propidium iodide (Sigma) according to manufacturer's specifications. Rapamycin rescue assays were performed in the presence of 20 ng/ml Rapamycin or vehicle control.

### Transcriptional reporter assay

Cells were treated with lentivirus as described above, and were plated into a 24-well plate, followed by transfection by lipofectamine 2000 (Invitrogen) according to the manufacturer's instruction. Each transfection contained 800 ng of TOPflash-luc or FOPflash-luc, and 5 ng of phRL-Luc. Cell extracts were prepared 48 hrs post-transfection, and the luciferase activity was measured using Dual Luciferase Reporter Assay System (Promega) according to the manufacturer's instruction. Luciferase activity was read on a BD Monolight 3010 Luminometer. All data points presented are the average measurement of three independent transfections.

### Soft agar growth assay and ROS assay

For growth assay, 10^4^ cells suspended in 0.35% agarose solution were poured over hard-bottomed agar (0.6%) previously solidified in 6-well plates. Cells were cultured in a humidified atmosphere with 5% CO2 at 37°C for 3–4 weeks, and then colonies were counted. Soft agar growth rescue assays were performed in the presence of 10 mM NAC or vehicle control added to the top layer mix at the time of plating.

For ROS assay, 10^5^ cells were seeded between top agar layer and bottom agar layer for 16 hrs, and then 1 ml of complete medium containing 20 µM of DHE was added onto the top agar layer. After incubation for 1 hr, the medium was aspirated and the top agar layer was carefully removed. Cells were processed for imaging with a Zeiss fluorescence microscope.

## Supporting Information

Figure S1 Characterization of *axn^127^* mutation. 3′ cDNA sequence of *axn* gene in *127* mutant is determined by 3′ RACE. Exon 10 of *axn^127^* is linked to a heterochromatin sequence (underlined sequence) instead of Exon 11 in *127* mutant (A and C). This change causes a deletion of part of the DIX domain at the C terminal of *Axn* protein (B and D). *127* mutant significantly increased Armadillo protein levels (E). (F–J) Eye discs with heat shock induced Flip-out clones shown in Fig. 2F–L. GFP marks the cells with Gal4 activation, which can drive the indicated RNAi and protein expression. Red and blue channels indicate cell death (caspase 3 staining) and photoreceptor differentiation (Elav staining), respectively.
(JPG)

Figure S2 Weak or strong *axn* alleles cause different effects on cell fate determination and apoptosis in eye and wing discs. In eye discs, *rbf*, *axn^127^*, or *rbf axn^127^* mutations do not have obvious effects on photoreceptor differentiation reflected by ELAV staining (A–C), while *axn^EY^*, or *rbf axn^EY^* mutations block photoreceptor differentiation (D–E). In wing discs, *rbf*, *axn^127^*, or *rbf axn^127^* mutations do not have obvious effects on wing margin cell fate determination reflected by Sens staining (F–H), while *axn^EY^*, or *rbf axn^EY^* mutations cause ectopic expression of Sens in wing pouch (I–J). *rbf axn^EY^* mutations induce synergistic apoptosis in wing discs (K–M), and Quantification of C3 levels is shown in panel N. *axn^S^* or *axn^EY^* mutation increases EGFR signaling activities in wing pouch reflected by pERK level and aos-lacz expression (O–P′).
(JPG)

Figure S3 Increased growth in *axn* mutant cells. *wt or axn* mutant clones and the corresponding wild type (wt) twin spots derived from the two daughter cells of a cell division are marked with absence of GFP and bright GFP respectively (A–C). wt mosaic clones have similar sizes with their twin spots (A), while both *axn^127^* and *axn^S^* mutant clones are significantly larger than their twin spots (B–C), and the ratio between mutant clones and twin spots are quantified in (D). Due to the suppression of differentiation by *axn^S^*, *axn^S^* clones in the whole discs and *wt* or *axn^127^* clones anterior to the MF are used for quantification. PCNA-GFP expression is upregulated in *axn^127^* mutant clones anterior to MF and in *axn^EY^* mutant clones located in different parts of the discs (E–F′). E2f1 protein is upregulated in *axn^EY^* mutant clones (G-G′). BrdU incorporation is increased in *axn^S^* mutant clones (H-H′).
(JPG)

Figure S4 (A–B), Eye discs with *axn^S^ rbf* and *axn^S^ rbf rheb* mutant clones in Minute background were shown. The mutant clones were marked by the absence of GFP signal. The ratios of clone region area verses the whole eye disc area were quantified and shown in (C). There is no significant difference in the relatively amount of mutant clone areas between the *axn^S^ rbf Minute* and the *axn^S^ rbf rheb Minute* eye discs.
(JPG)

Figure S5 Inactivation of APC and Rb shows synergistic cell death effect in Du145 and HC116 cells with additional shRNA constructs. Du145 cells with APC knockdown construct shAPC-2 showed higher level of Wnt reporter activity in TOP luciferase assay (A). APC knockdown enhanced cell death (B), decreased viable cell numbers (C) and inhibited colony growth in soft agar assay (D). In HCT116 cells, Rb knockdown construct shRb-1 decreased the Rb protein level (E). (F–G) The effect of knockdown Rb and APC on Wnt signaling activity detected by TOP luciferase assay (F) and apoptosis detected by Annexin V and PI staining in HCT 116 cells.
(JPG)

Table S1 Genes up- or downregulated (>2 folds, P<0.05) in *axn^127^* mutants as compared to WT control L3 larvae.
(PDF)

Table S2 Gene Ontology (GO) term enrichment of genes that are significantly up- or downregulated (>2 folds, P<0.05) in *axn^127^* mutant L3 larvae. GO terms that significantly enriched (P<0.0001) are shown. Consistent with the energy deficiency of the *axn^127^* mutant, genes involved carbohydrate and lipid metabolism are significantly downregulated, while genes related to stress or stimulus response are significantly upregulated. Consistent with *axn* mutation increasing *Wg* signaling activities, genes related to morphogenesis and signal transduction are upregulated in *axn^127^* mutant.
(PDF)

## References

[1] Knudsen ES, Knudsen KE (2008) Tailoring to RB: tumour suppressor status and therapeutic response. Nat Rev Cancer 8: 714–724. 19143056 10.1038/nrc2401 PMC2914856

[2] Burkhart DL, Sage J (2008) Cellular mechanisms of tumour suppression by the retinoblastoma gene. Nat Rev Cancer 8: 671–682. 18650841 10.1038/nrc2399 PMC6996492

[3] Gordon GM, Du W (2011) Targeting Rb inactivation in cancers by synthetic lethality. Am J Cancer Res 1: 773–786. 21814623 PMC3147291

[4] van den Heuvel S, Dyson NJ (2008) Conserved functions of the pRB and E2F families. Nat Rev Mol Cell Biol 9: 713–724. 18719710 10.1038/nrm2469

[5] Gordon GM, Du W (2011) Conserved RB functions in development and tumor suppression. Protein Cell 2: 864–878. 22180086 10.1007/s13238-011-1117-z PMC3271014

[6] Steele L, Sukhanova MJ, Xu J, Gordon GM, Huang Y, et al (2009) Retinoblastoma family protein promotes normal R8-photoreceptor differentiation in the absence of rhinoceros by inhibiting dE2F1 activity. Dev Biol 335: 228–236. 19744473 10.1016/j.ydbio.2009.09.004 PMC2763947

[7] Sukhanova MJ, Steele LJ, Zhang T, Gordon GM, Du W (2011) RBF and Rno promote photoreceptor differentiation onset through modulating EGFR signaling in the Drosophila developing eye. Dev Biol 359: 190–198. 21920355 10.1016/j.ydbio.2011.08.018 PMC3202672

[8] Tanaka-Matakatsu M, Xu J, Cheng L, Du W (2009) Regulation of apoptosis of rbf mutant cells during Drosophila development. Dev Biol 326: 347–356. 19100727 10.1016/j.ydbio.2008.11.035 PMC2634822

[9] Gordon GM, Zhang T, Zhao J, Du W (2013) Deregulated G1-S control and energy stress contribute to the synthetic-lethal interactions between inactivation of RB and TSC1 or TSC2. J Cell Sci 126: 2004–2013. 23447678 10.1242/jcs.121301 PMC3666254

[10] Li B, Gordon GM, Du CH, Xu J, Du W (2010) Specific killing of Rb mutant cancer cells by inactivating TSC2. Cancer Cell 17: 469–480. 20478529 10.1016/j.ccr.2010.03.019 PMC2873973

[11] Hsieh TC, Nicolay BN, Frolov MV, Moon NS (2010) Tuberous sclerosis complex 1 regulates dE2F1 expression during development and cooperates with RBF1 to control proliferation and survival. PLoS Genet 6: e1001071. 20808898 10.1371/journal.pgen.1001071 PMC2924346

[12] Danos AM, Liao Y, Li X, Du W (2013) Functional inactivation of Rb sensitizes cancer cells to TSC2 inactivation induced cell death. Cancer Lett 328: 36–43. 23022476 10.1016/j.canlet.2012.09.016 PMC3494767

[13] Potter CJ, Huang H, Xu T (2001) Drosophila Tsc1 functions with Tsc2 to antagonize insulin signaling in regulating cell growth, cell proliferation, and organ size. Cell 105: 357–368. 11348592 10.1016/s0092-8674(01)00333-6

[14] Tapon N, Ito N, Dickson BJ, Treisman JE, Hariharan IK (2001) The Drosophila tuberous sclerosis complex gene homologs restrict cell growth and cell proliferation. Cell 105: 345–355. 11348591 10.1016/s0092-8674(01)00332-4

[15] Nicolay BN, Gameiro PA, Tschop K, Korenjak M, Heilmann AM, et al (2013) Loss of RBF1 changes glutamine catabolism. Genes Dev 27: 182–196. 23322302 10.1101/gad.206227.112 PMC3566311

[16] Cui M, Cohen ML, Teng C, Han M (2013) The tumor suppressor Rb critically regulates starvation-induced stress response in C. elegans. Curr Biol 23: 975–980. 23664972 10.1016/j.cub.2013.04.046 PMC3728909

[17] Reynolds MR, Lane AN, Robertson B, Kemp S, Liu Y, et al (2013) Control of glutamine metabolism by the tumor suppressor Rb. Oncogene 33: 556–66 doi: 10.1038/onc.2012.635 23353822 10.1038/onc.2012.635 PMC3918885

[18] Moon NS, Di Stefano L, Dyson N (2006) A gradient of epidermal growth factor receptor signaling determines the sensitivity of rbf1 mutant cells to E2F-dependent apoptosis. Mol Cell Biol 26: 7601–7615. 16954388 10.1128/MCB.00836-06 PMC1636876

[19] Du W (2000) Suppression of the rbf null mutants by a de2f1 allele that lacks transactivation domain. Development 127: 367–379. 10603353 10.1242/dev.127.2.367

[20] Hamada F, Tomoyasu Y, Takatsu Y, Nakamura M, Nagai S, et al (1999) Negative regulation of Wingless signaling by D-axin, a Drosophila homolog of axin. Science 283: 1739–1742. 10073940 10.1126/science.283.5408.1739

[21] Lee JD, Treisman JE (2001) The role of Wingless signaling in establishing the anteroposterior and dorsoventral axes of the eye disc. Development 128: 1519–1529. 11290291 10.1242/dev.128.9.1519

[22] Abdou M, Peng C, Huang J, Zyaan O, Wang S, et al (2011) Wnt signaling cross-talks with JH signaling by suppressing Met and gce expression. PLoS One 6: e26772. 22087234 10.1371/journal.pone.0026772 PMC3210751

[23] Baonza A, Freeman M (2002) Control of Drosophila eye specification by Wingless signalling. Development 129: 5313–5322. 12403704 10.1242/dev.00096

[24] Nolo R, Abbott LA, Bellen HJ (2001) Drosophila Lyra mutations are gain-of-function mutations of senseless. Genetics 157: 307–315. 11139511 10.1093/genetics/157.1.307 PMC1461469

[25] Bergmann A, Agapite J, McCall K, Steller H (1998) The Drosophila gene hid is a direct molecular target of Ras-dependent survival signaling. Cell 95: 331–341. 9814704 10.1016/s0092-8674(00)81765-1

[26] Kurada P, White K (1998) Ras promotes cell survival in Drosophila by downregulating hid expression. Cell 95: 319–329. 9814703 10.1016/s0092-8674(00)81764-x

[27] Yang L, Baker NE (2003) Cell cycle withdrawal, progression, and cell survival regulation by EGFR and its effectors in the differentiating Drosophila eye. Dev Cell 4: 359–369. 12636917 10.1016/s1534-5807(03)00059-5

[28] Dominguez M, Wasserman JD, Freeman M (1998) Multiple functions of the EGF receptor in Drosophila eye development. Curr Biol 8: 1039–1048. 9768358 10.1016/s0960-9822(98)70441-5

[29] Choo AY, Kim SG, Vander Heiden MG, Mahoney SJ, Vu H, et al (2010) Glucose addiction of TSC null cells is caused by failed mTORC1-dependent balancing of metabolic demand with supply. Mol Cell 38: 487–499. 20513425 10.1016/j.molcel.2010.05.007 PMC2896794

[30] Du W, Dyson N (1999) The role of RBF in the introduction of G1 regulation during Drosophila embryogenesis. EMBO J 18: 916–925. 10022834 10.1093/emboj/18.4.916 PMC1171184

[31] Bilak A, Su TT (2009) Regulation of Drosophila melanogaster pro-apoptotic gene hid. Apoptosis 14: 943–949. 19554451 10.1007/s10495-009-0374-2 PMC3373429

[32] Steller H (2008) Regulation of apoptosis in Drosophila. Cell Death Differ 15: 1132–1138. 18437164 10.1038/cdd.2008.50

[33] Moon NS, Frolov MV, Kwon EJ, Di Stefano L, Dimova DK, et al (2005) Drosophila E2F1 has context-specific pro- and antiapoptotic properties during development. Dev Cell 9: 463–475. 16198289 10.1016/j.devcel.2005.08.015

[34] Bookstein R, Shew JY, Chen PL, Scully P, Lee WH (1990) Suppression of tumorigenicity of human prostate carcinoma cells by replacing a mutated RB gene. Science 247: 712–715. 2300823 10.1126/science.2300823

[35] Gope R, Christensen MA, Thorson A, Lynch HT, Smyrk T, et al (1990) Increased expression of the retinoblastoma gene in human colorectal carcinomas relative to normal colonic mucosa. J Natl Cancer Inst 82: 310–314. 2405172 10.1093/jnci/82.4.310

[36] Morris EJ, Ji JY, Yang F, Di Stefano L, Herr A, et al (2008) E2F1 represses beta-catenin transcription and is antagonized by both pRB and CDK8. Nature 455: 552–556. 18794899 10.1038/nature07310 PMC3148807

[37] Sansom OJ, Reed KR, Hayes AJ, Ireland H, Brinkmann H, et al (2004) Loss of Apc in vivo immediately perturbs Wnt signaling, differentiation, and migration. Genes Dev 18: 1385–1390. 15198980 10.1101/gad.287404 PMC423189

[38] Benchabane H, Ahmed Y (2009) The adenomatous polyposis coli tumor suppressor and Wnt signaling in the regulation of apoptosis. Adv Exp Med Biol 656: 75–84. 19928354 10.1007/978-1-4419-1145-2_7 PMC3066060

[39] Qian Z, Chen L, Fernald AA, Williams BO, Le Beau MM (2008) A critical role for Apc in hematopoietic stem and progenitor cell survival. J Exp Med 205: 2163–2175. 18725524 10.1084/jem.20080578 PMC2526209

[40] Biechele TL, Kulikauskas RM, Toroni RA, Lucero OM, Swift RD, et al (2012) Wnt/beta-catenin signaling and AXIN1 regulate apoptosis triggered by inhibition of the mutant kinase BRAFV600E in human melanoma. Sci Signal 5: ra3. 22234612 10.1126/scisignal.2002274 PMC3297477

[41] Stott FJ, Bates S, James MC, McConnell BB, Starborg M, et al (1998) The alternative product from the human CDKN2A locus, p14(ARF), participates in a regulatory feedback loop with p53 and MDM2. EMBO J 17: 5001–5014. 9724636 10.1093/emboj/17.17.5001 PMC1170828

[42] Yang W, Soares J, Greninger P, Edelman EJ, Lightfoot H, et al (2013) Genomics of Drug Sensitivity in Cancer (GDSC): a resource for therapeutic biomarker discovery in cancer cells. Nucleic Acids Res 41: D955–961. 23180760 10.1093/nar/gks1111 PMC3531057

[43] Zhao J, Zhang Z, Liao Y, Du W (2014) Mutation of the retinoblastoma tumor suppressor gene sensitizes cancers to mitotic inhibitor induced cell death. Am J Cancer Res 4: 42–52. 24482737 PMC3902231

[44] Benjamin D, Colombi M, Moroni C, Hall MN (2011) Rapamycin passes the torch: a new generation of mTOR inhibitors. Nat Rev Drug Discov 10: 868–880. 22037041 10.1038/nrd3531

[45] Inoki K, Ouyang H, Zhu T, Lindvall C, Wang Y, et al (2006) TSC2 integrates Wnt and energy signals via a coordinated phosphorylation by AMPK and GSK3 to regulate cell growth. Cell 126: 955–968. 16959574 10.1016/j.cell.2006.06.055

[46] Castilho RM, Squarize CH, Chodosh LA, Williams BO, Gutkind JS (2009) mTOR mediates Wnt-induced epidermal stem cell exhaustion and aging. Cell Stem Cell 5: 279–289. 19733540 10.1016/j.stem.2009.06.017 PMC2939833

[47] Valvezan AJ, Zhang F, Diehl JA, Klein PS (2012) Adenomatous polyposis coli (APC) regulates multiple signaling pathways by enhancing glycogen synthase kinase-3 (GSK-3) activity. J Biol Chem 287: 3823–3832. 22184111 10.1074/jbc.M111.323337 PMC3281685

[48] Liu H, Remedi MS, Pappan KL, Kwon G, Rohatgi N, et al (2009) Glycogen synthase kinase-3 and mammalian target of rapamycin pathways contribute to DNA synthesis, cell cycle progression, and proliferation in human islets. Diabetes 58: 663–672. 19073772 10.2337/db07-1208 PMC2646065

[49] Buller CL, Loberg RD, Fan MH, Zhu Q, Park JL, et al (2008) A GSK-3/TSC2/mTOR pathway regulates glucose uptake and GLUT1 glucose transporter expression. Am J Physiol Cell Physiol 295: C836–843. 18650261 10.1152/ajpcell.00554.2007 PMC2544442

[50] Shin S, Wolgamott L, Yu Y, Blenis J, Yoon SO (2011) Glycogen synthase kinase (GSK)-3 promotes p70 ribosomal protein S6 kinase (p70S6K) activity and cell proliferation. Proc Natl Acad Sci U S A 108: E1204–1213. 22065737 10.1073/pnas.1110195108 PMC3223461

[51] Hirabayashi S, Baranski TJ, Cagan RL (2013) Transformed drosophila cells evade diet-mediated insulin resistance through wingless signaling. Cell 154: 664–675. 23911328 10.1016/j.cell.2013.06.030 PMC3800019

[52] Gao X, Pan D (2001) TSC1 and TSC2 tumor suppressors antagonize insulin signaling in cell growth. Genes Dev 15: 1383–1392. 11390358 10.1101/gad.901101 PMC312704

[53] Lee JH, Koh H, Kim M, Park J, Lee SY, et al (2006) JNK pathway mediates apoptotic cell death induced by tumor suppressor LKB1 in Drosophila. Cell Death Differ 13: 1110–1122. 16273080 10.1038/sj.cdd.4401790

[54] Takacs CM, Baird JR, Hughes EG, Kent SS, Benchabane H, et al (2008) Dual positive and negative regulation of wingless signaling by adenomatous polyposis coli. Science 319: 333–336. 18202290 10.1126/science.1151232

[55] Fan Y, Lee TV, Xu D, Chen Z, Lamblin AF, et al (2010) Dual roles of Drosophila p53 in cell death and cell differentiation. Cell Death Differ 17: 912–921. 19960025 10.1038/cdd.2009.182 PMC3014827

[56] Thacker SA, Bonnette PC, Duronio RJ (2003) The contribution of E2F-regulated transcription to Drosophila PCNA gene function. Curr Biol 13: 53–58. 12526745 10.1016/s0960-9822(02)01400-8

[57] Nolo R, Abbott LA, Bellen HJ (2000) Senseless, a Zn finger transcription factor, is necessary and sufficient for sensory organ development in Drosophila. Cell 102: 349–362. 10975525 10.1016/s0092-8674(00)00040-4

[58] Ryoo HD, Gorenc T, Steller H (2004) Apoptotic cells can induce compensatory cell proliferation through the JNK and the Wingless signaling pathways. Dev Cell 7: 491–501. 15469838 10.1016/j.devcel.2004.08.019

[59] Zhao X, Feng D, Wang Q, Abdulla A, Xie XJ, et al (2012) Regulation of lipogenesis by cyclin-dependent kinase 8-mediated control of SREBP-1. J Clin Invest 122: 2417–2427. 22684109 10.1172/JCI61462 PMC3386818

[60] Boyle EI, Weng S, Gollub J, Jin H, Botstein D, et al (2004) GO::TermFinder–open source software for accessing Gene Ontology information and finding significantly enriched Gene Ontology terms associated with a list of genes. Bioinformatics 20: 3710–3715. 15297299 10.1093/bioinformatics/bth456 PMC3037731

