# Supplementary figures and images for: Hyperactivated Wnt Signaling Induces Synthetic Lethal Interaction with Rb Inactivation by Elevating TORC1 Activities
Source: PLoS Genet. 2014 May 8;10(5):e1004357. doi: 10.1371/journal.pgen.1004357 (PMC4014429; doi:10.1371/journal.pgen.1004357)

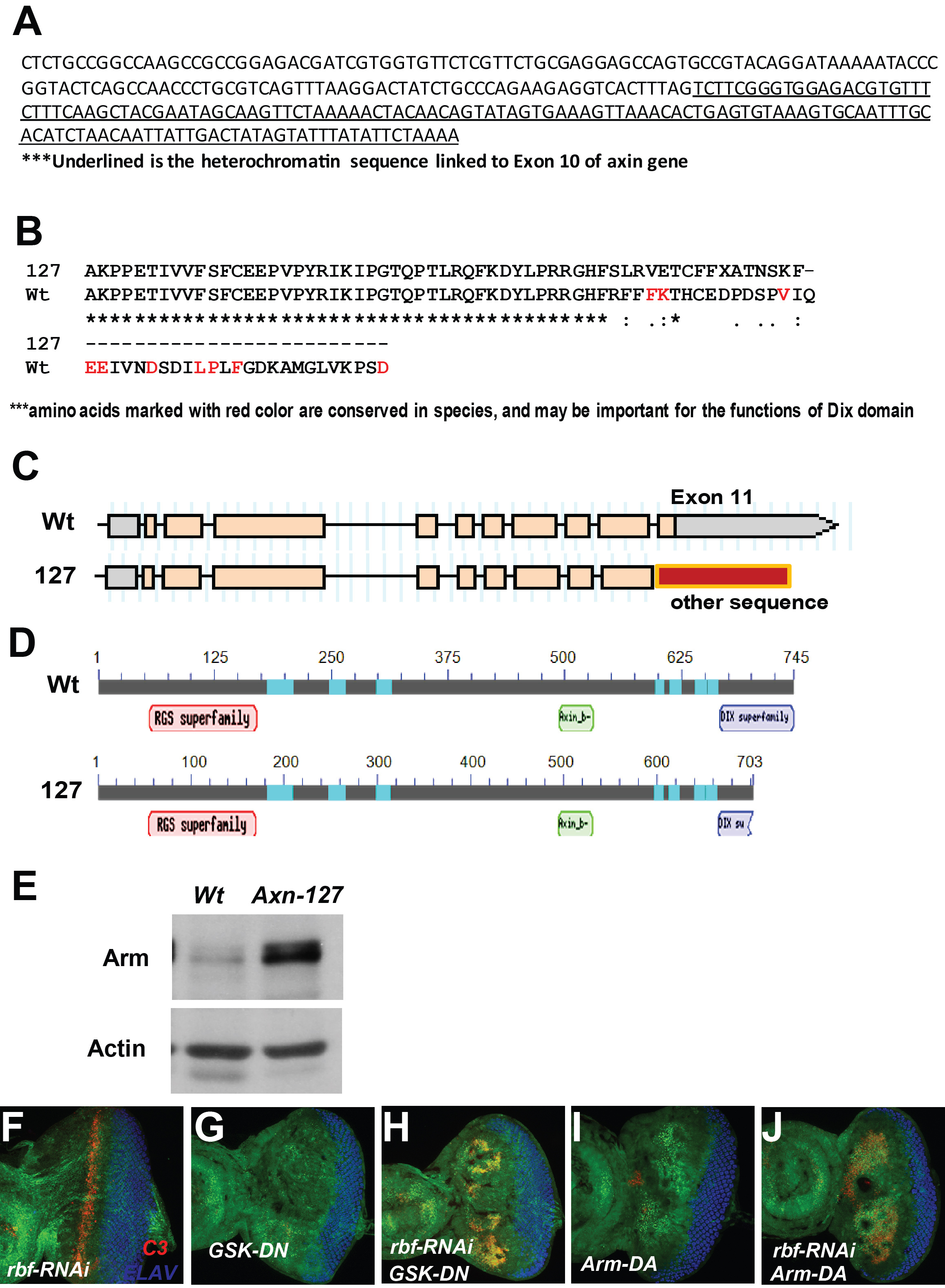

Supplement: Figure S1 — Characterization of axn127 mutation. 3′ cDNA sequence of axn gene in 127 mutant is determined by 3′ RACE. Exon 10 of axn127 is linked to a heterochromatin sequence (underlined sequence) instead of Exon 11 in 127 mutant (A and C). This change causes a deletion of part of the DIX domain at the C terminal of Axn protein (B and D). 127 mutant significantly increased Armadillo protein levels (E). (F–J) Eye discs with heat shock induced Flip-out clones shown in Fig. 2F–L. GFP marks the cells with Gal4 activation, which can drive the indicated RNAi and protein expression. Red and blue channels indicate cell death (caspase 3 staining) and photoreceptor differentiation (Elav staining), respectively. (JPG) [file pgen.1004357.s001.jpg]

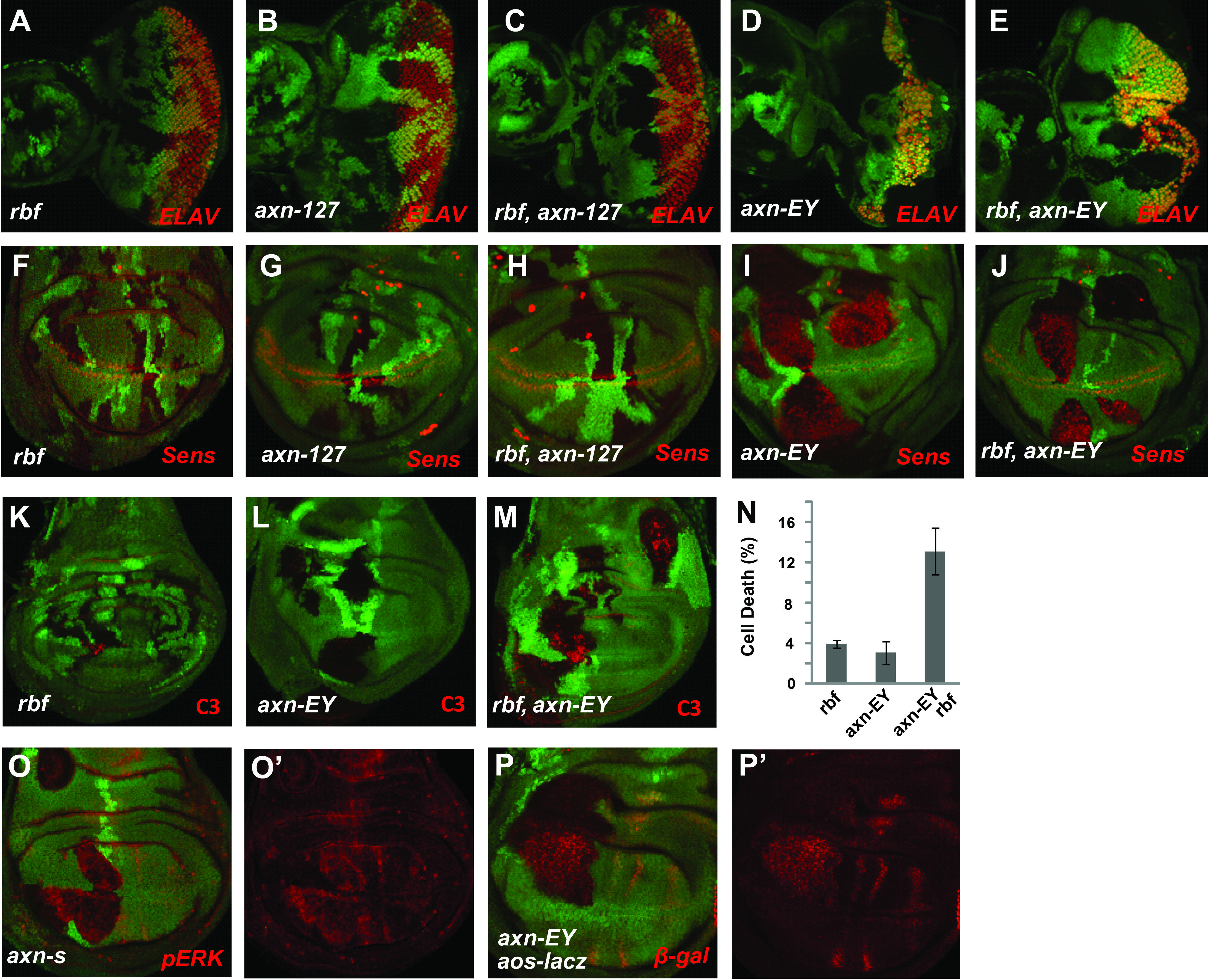

Supplement: Figure S2 — Weak or strong axn alleles cause different effects on cell fate determination and apoptosis in eye and wing discs. In eye discs, rbf, axn127, or rbf axn127 mutations do not have obvious effects on photoreceptor differentiation reflected by ELAV staining (A–C), while axnEY, or rbf axnEY mutations block photoreceptor differentiation (D–E). In wing discs, rbf, axn127, or rbf axn127 mutations do not have obvious effects on wing margin cell fate determination reflected by Sens staining (F–H), while axnEY, or rbf axnEY mutations cause ectopic expression of Sens in wing pouch (I–J). rbf axnEY mutations induce synergistic apoptosis in wing discs (K–M), and Quantification of C3 levels is shown in panel N. axnS or axnEY mutation increases EGFR signaling activities in wing pouch reflected by pERK level and aos-lacz expression (O–P′). (JPG) [file pgen.1004357.s002.jpg]

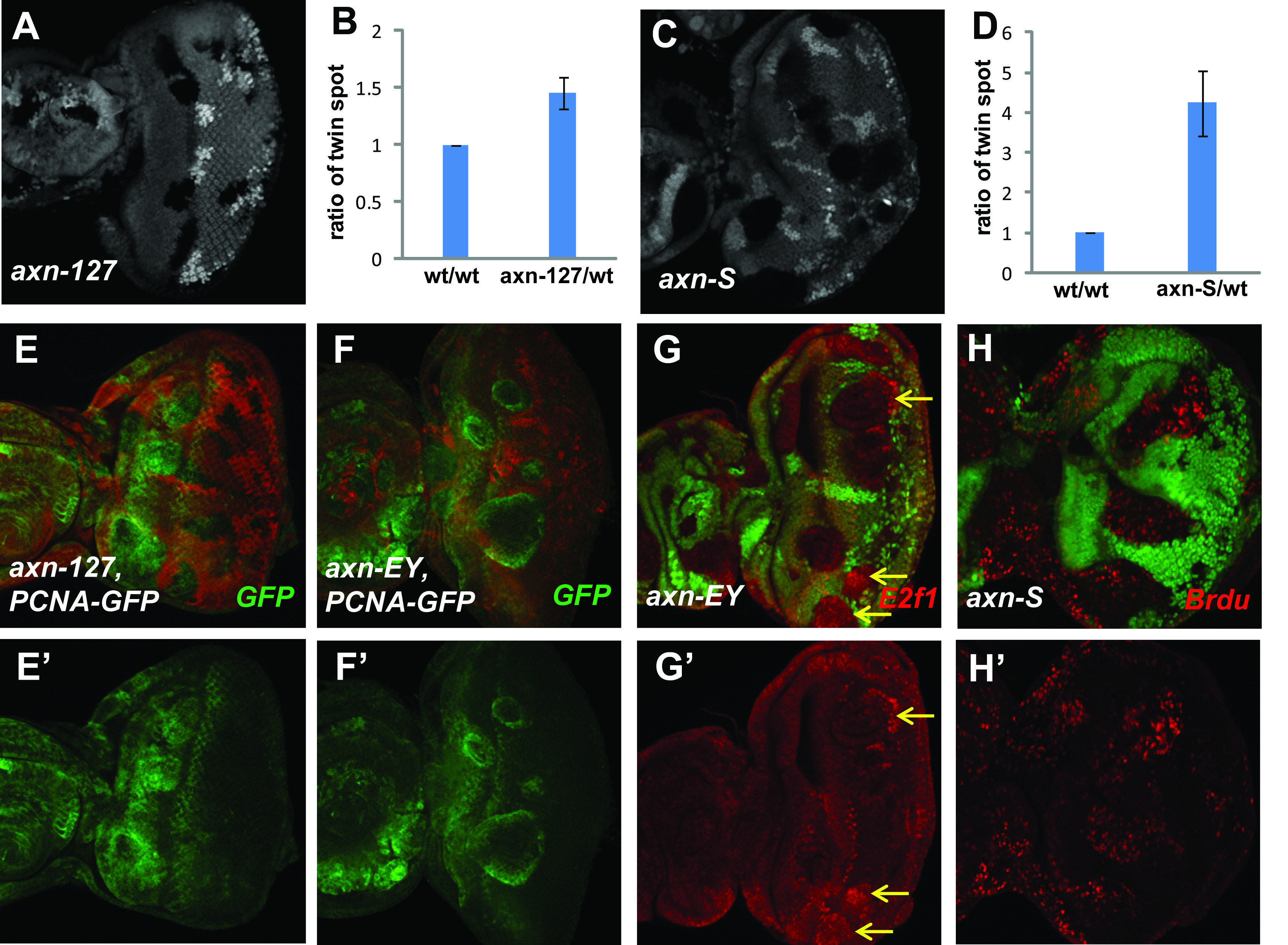

Supplement: Figure S3 — Increased growth in axn mutant cells. wt or axn mutant clones and the corresponding wild type (wt) twin spots derived from the two daughter cells of a cell division are marked with absence of GFP and bright GFP respectively (A–C). wt mosaic clones have similar sizes with their twin spots (A), while both axn127 and axnS mutant clones are significantly larger than their twin spots (B–C), and the ratio between mutant clones and twin spots are quantified in (D). Due to the suppression of differentiation by axnS, axnS clones in the whole discs and wt or axn127 clones anterior to the MF are used for quantification. PCNA-GFP expression is upregulated in axn127 mutant clones anterior to MF and in axnEY mutant clones located in different parts of the discs (E–F′). E2f1 protein is upregulated in axnEY mutant clones (G-G′). BrdU incorporation is increased in axnS mutant clones (H-H′). (JPG) [file pgen.1004357.s003.jpg]

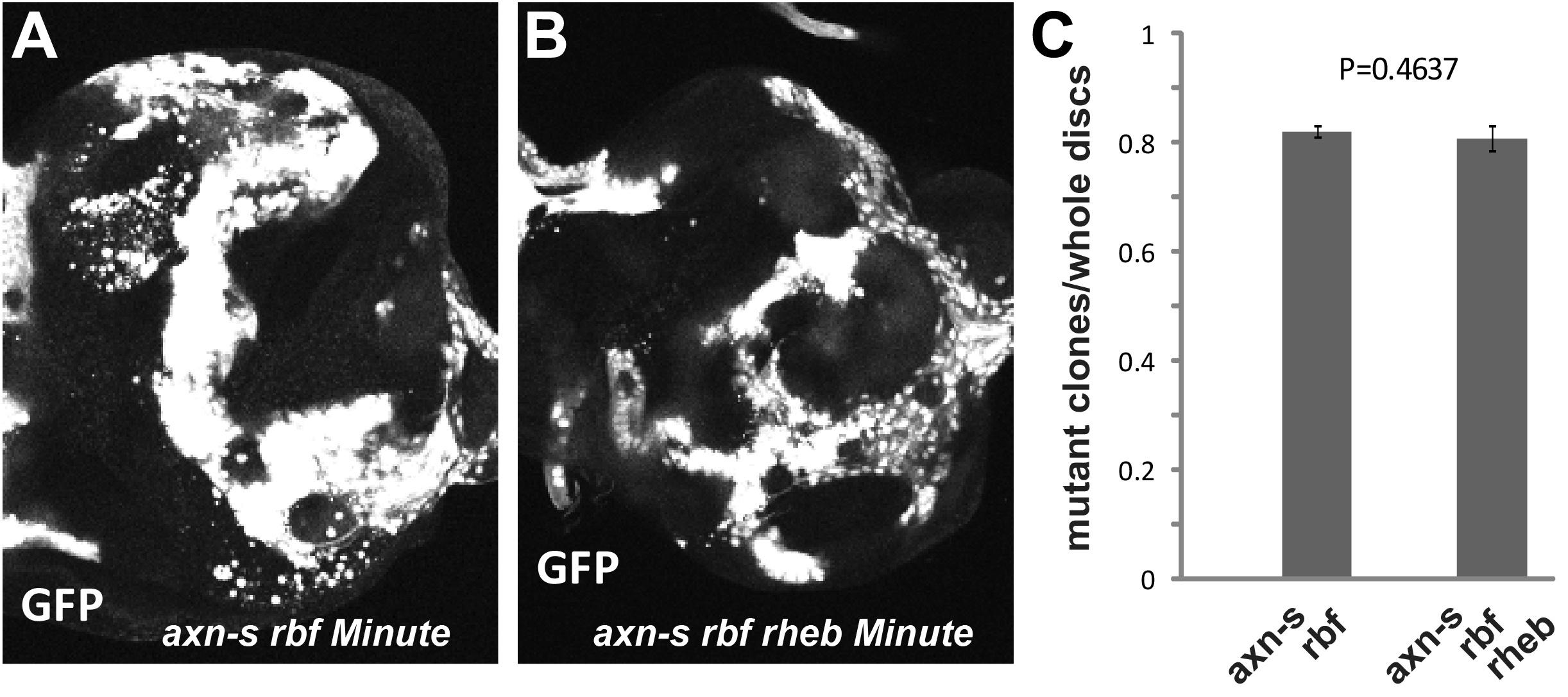

Supplement: Figure S4 — (A–B), Eye discs with axnS rbf and axnS rbf rheb mutant clones in Minute background were shown. The mutant clones were marked by the absence of GFP signal. The ratios of clone region area verses the whole eye disc area were quantified and shown in (C). There is no significant difference in the relatively amount of mutant clone areas between the axnS rbf Minute and the axnS rbf rheb Minute eye discs. (JPG) [file pgen.1004357.s004.jpg]

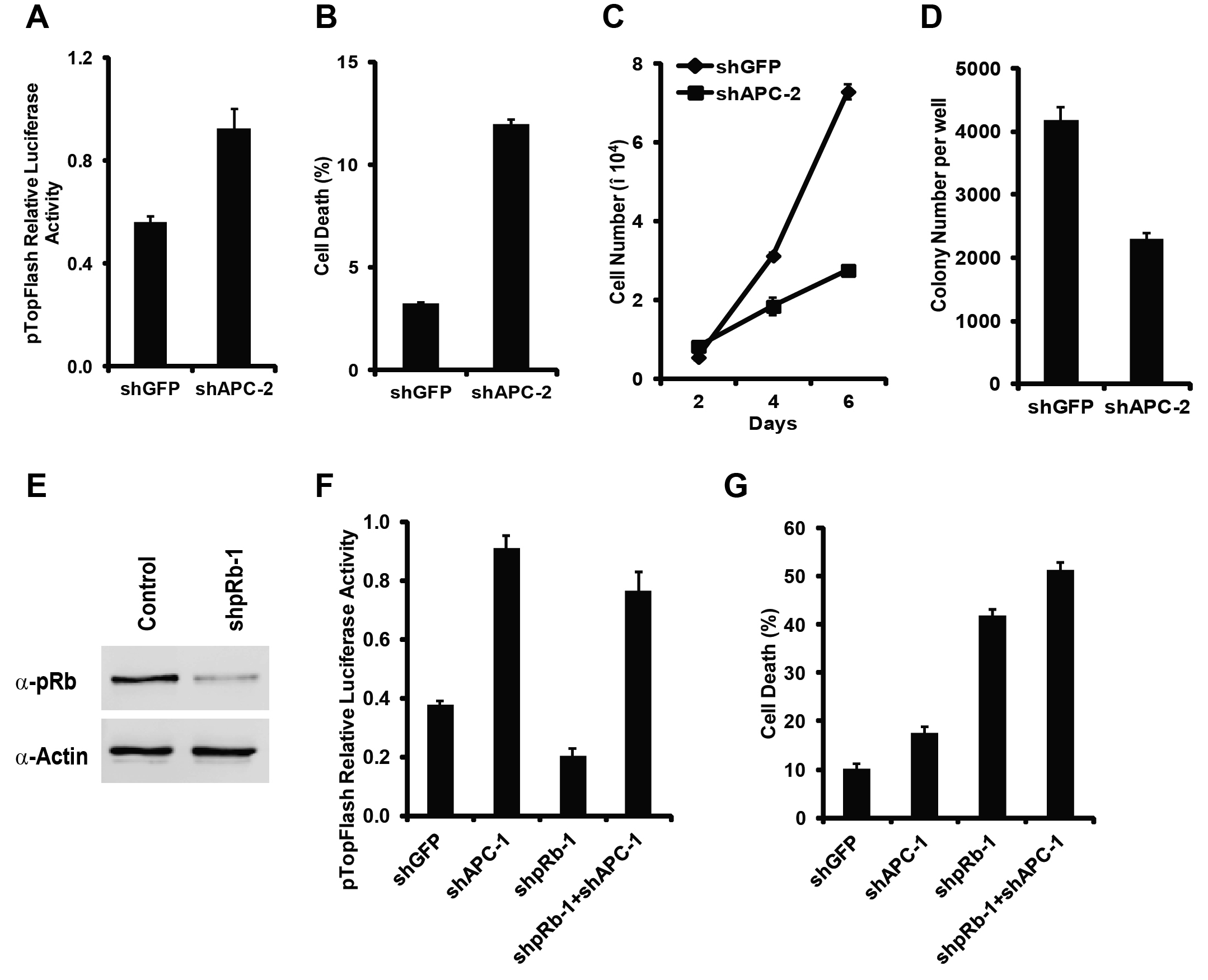

Supplement: Figure S5 — Inactivation of APC and Rb shows synergistic cell death effect in Du145 and HC116 cells with additional shRNA constructs. Du145 cells with APC knockdown construct shAPC-2 showed higher level of Wnt reporter activity in TOP luciferase assay (A). APC knockdown enhanced cell death (B), decreased viable cell numbers (C) and inhibited colony growth in soft agar assay (D). In HCT116 cells, Rb knockdown construct shRb-1 decreased the Rb protein level (E). (F–G) The effect of knockdown Rb and APC on Wnt signaling activity detected by TOP luciferase assay (F) and apoptosis detected by Annexin V and PI staining in HCT 116 cells. (JPG) [file pgen.1004357.s005.jpg]
