# Supplementary material for: Hyperactivated Wnt Signaling Induces Synthetic Lethal Interaction with Rb Inactivation by Elevating TORC1 Activities
Source: PLoS Genet. 2014 May 8;10(5):e1004357. doi: 10.1371/journal.pgen.1004357 (PMC4014429; doi:10.1371/journal.pgen.1004357)
Supplement: Table S1 — Genes up- or downregulated (>2 folds, P<0.05) in axn127 mutants as compared to WT control L3 larvae. (PDF) [file pgen.1004357.s006.pdf]

Supplemental Table S1

Go term	P-Value
**downregulated in axn-127 mutant**	
**metabolism**	
GO:0044710 single-organism metabolic process	2.87E-15
GO:0008152 metabolic process	2.56E-12
GO:0005975 carbohydrate metabolic process	5.69E-06
GO:0006629 lipid metabolic process	6.10E-06
**oxidation-reduction**	
GO:0055114 oxidation-reduction process	1.47E-09
**upregulated in axn-127 mutant**	
**morphogenesis, development and differention**	
GO:0035107 appendage morphogenesis	3.40E-09
GO:0048736 appendage development	5.80E-09
GO:0048563 post-embryonic organ morphogenesis	9.53E-09
GO:0007560 imaginal disc morphogenesis	9.53E-09
GO:0007476 imaginal disc-derived wing morphogenesis	9.97E-09
GO:0048569 post-embryonic organ development	9.98E-09
GO:0035220 wing disc development	1.13E-08
GO:0035120 post-embryonic appendage morphogenesis	1.33E-08
GO:0035114 imaginal disc-derived appendage morphogenesis	1.63E-08
GO:0007472 wing disc morphogenesis	2.33E-08
GO:0048737 imaginal disc-derived appendage development	2.74E-08
GO:0007444 imaginal disc development	3.70E-08
GO:0006928 cellular component movement	4.20E-08
GO:0048707 instar larval or pupal morphogenesis	4.77E-08
GO:0009886 post-embryonic morphogenesis	6.64E-08
GO:0009791 post-embryonic development	7.87E-08
GO:0002165 instar larval or pupal development	1.27E-07
GO:0000902 cell morphogenesis	3.11E-07
GO:0010646 regulation of cell communication	5.51E-07
GO:0007552 metamorphosis	7.06E-07
GO:0050793 regulation of developmental process	1.33E-06
GO:0050794 regulation of cellular process	4.55E-06
GO:0007283 spermatogenesis	1.87E-05
GO:0048232 male gamete generation	2.15E-05
GO:0032989 cellular component morphogenesis	2.33E-05

GO:0048468 cell development	2.45E-05
GO:0010648 negative regulation of cell communication	3.22E-05
**signaling pathways**	
GO:0023051 regulation of signaling	2.28E-07
GO:0009966 regulation of signal transduction	5.34E-07
GO:0009968 negative regulation of signal transduction	7.38E-06
**response to stimulus or stress**	
GO:0048585 negative regulation of response to stimulus	3.59E-05
GO:0048583 regulation of response to stimulus	3.90E-05
**other**	
GO:0050789 regulation of biological process	4.69E-05
GO:0044763 single-organism cellular process	5.51E-05
