# Supplementary material for: Hyperactivated Wnt Signaling Induces Synthetic Lethal Interaction with Rb Inactivation by Elevating TORC1 Activities
Source: PLoS Genet. 2014 May 8;10(5):e1004357. doi: 10.1371/journal.pgen.1004357 (PMC4014429; doi:10.1371/journal.pgen.1004357)
Supplement: Table S2 — Gene Ontology (GO) term enrichment of genes that are significantly up- or downregulated (>2 folds, P<0.05) in axn127 mutant L3 larvae. GO terms that significantly enriched (P<0.0001) are shown. Consistent with the energy deficiency of the axn127 mutant, genes involved carbohydrate and lipid metabolism are significantly downregulated, while genes related to stress or stimulus response are significantly upregulated. Consistent with axn mutation increasing Wg signaling activities, genes related to morphogenesis and signal transduction are upregulated in axn127 mutant. (PDF) [file pgen.1004357.s007.pdf]

Supplemental Table S2

genes downregulated in 127	genes upregulated in 127
CG32280	2mit
1-Sep	5-HT7
Aats-glupro	Abl
Aats-gly	achi /// vis
Aats-pro	acj6
abd-A	Acp95EF
abo	Acsl
Acon	Actbeta
Acp65Aa	Actn3
Ada	ACXA
ade5	ACXC
Adgf-C	Adar
Adh /// Adhr	AGO1
adp	alc
Ady43A	alph
Ag5r2	alphaTub84D /// Tm1
Ahcy13	amon
Ahcy89E	Amun
Alg10	And /// CG17770
alpha4GT1	Apc2
alpha-Cat	app
alpha-Est4	apt
alpha-Est9	Arc1
alphaTub85E	CG10102
Aly	aret
Amy-d /// Amy-p	ari-1
ana	Arp14D
Ance-4	Asph
Ance-5	Atac1
angel	Atg8b
anon-67Ea /// hay	Atpalpha
Aos1	AttA
Aph-4	AttB
Aplip1	AttC
AR-2	B4
aralar1	B52
Arc42	bam
Ard1 /// CG42455	bbg
aret	be
arg	beat-IIIa /// CG34106
Ark	bel
Arp11	betaInt-nu
Arpc3A	bgm
Arr1	BicC

asparagine-synthetase
Asph
Ast
Atg7
Atg9
ATPsyn-b
ATPsyn-beta
ATPsyn-d
ATPsyn-gamma
att-ORFA /// att-ORFB
Atx-1 /// CG4542
Awh
Axn
b
B4
B52
ball
bap
beg
beta3GalTII
beta4GalNAcTB
betaCop
betaggt-II
betaTry
BG4
Bgb
bib
Bin1
Bka
BM-40-SPARC
Brd8
Brf
bsf
BtbVII
Btd
Bub3
bys
C1GalTA
Cad86C
cag
CAH1
CAH2
CalpA
CanA1
CanB
Catsup
cbt
bl
Bmcp
bnl
bol
br
brat
brp
BthD
Btk29A
btsz
bun
bur
bves
Bx
by
C3G
ca
cal1
CalpB
Calx
Camta
can
Caps
capu
cas
Cby
CCS
Cct2
Cda5
Cdc6
Cdep
Cdlc2
CecC
cenG1A
CG10039
CG10055
CG10062
CG10063
CG10068
CG10073
CG10086
CG10089
CG10202
CG10249
CG10298
CG10300
CG10307

Ccp84Ac
Ccp84Ae
Ccp84Af
CDase
Cdep
Cenp-C
CG10006
CG10026
CG10031
CG10131
CG10153
CG10178
CG10189
CG10195
CG10209
CG10214
CG10237
CG10311
CG10347
CG1041
CG10418
CG10424
CG10444
CG10467
CG10469
CG10476 /// CG18606
CG10477
CG10512
CG10513
CG10514
CG10566
CG10623
CG10628
CG10638
CG10639
CG10646
CG10681
CG10754
CG10764
CG10795
CG10799
CG10824 /// DsimGD20003
CG10825
CG10827
CG10904
CG10927
CG10933
CG10317
CG10320
CG10337
CG10383
CG10396
CG10433
CG10494
CG10508
CG10543
CG10588
CG10650
CG10694
CG10734
CG10752
CG10761
CG10777
CG10793
CG10809
CG10834
CG10841
CG10859
CG10869
CG10880
CG10899
CG10910
CG10911
CG10912
CG10918
CG10919
CG10920
CG10934
CG10943
CG10969
CG10970
CG11023
CG11030
CG11042
CG11043
CG11050
CG11052
CG11068
CG11093
CG11106
CG11125
CG11138
CG11165
CG30378

CG11029
CG1103
CG11073
CG11077
CG11095
CG11110
CG11123 /// DmirCG11123
CG11127
CG11131
CG11134
CG1115
CG11151
CG11170
CG11210
CG11257
CG11284
CG11300
CG11313
CG11345
CG11349
CG1136
CG11377
CG11382
CG11388
CG1139
CG11425
CG11438
CG11444
CG11459 /// DsimGD19566
CG11470
CG11534
CG11539
CG11576
CG11594
CG11601
CG1161
CG11638
CG11652
CG11668
CG11679
CG11695
CG11696
CG11710
CG11737
CG11752
CG11753
CG11788
CG11226
CG11262
CG11291
CG11298
CG11322
CG11327
CG11360
CG11362
CG1137
CG11373
CG11374
CG11384
CG11396
CG11404
CG11426
CG11448
CG1146
CG11462
CG11505
CG11523
CG11537
CG11563
CG11586
CG11658
CG11666
CG11671
CG11694
CG11697
CG11698
CG11703
CG11796 /// DsecCG11796
CG11873
CG11929
CG11964
CG11970
CG12007
CG12027
CG12038
CG12069
CG12075
CG12106
CG12128
CG12130 /// DpseGA11425
CG12147
CG12179
CG12184
CG12201

CG11820
CG11835
CG11836
CG11851
CG11875
CG11876
CG11882
CG11892
CG11911
CG11912 /// DsimCG11912
CG11926
CG11975
CG12006
CG12009
CG12012
CG12018
CG12024
CG12056
CG12057
CG12082
CG12093
CG12096
CG12116 /// DsecCG12116
CG12119
CG1213
CG12162
CG12170
CG12173
CG12203
CG12219
CG12253
CG12259
CG12264
CG12272
CG12279
CG1231
CG12375
CG1239
CG12404
CG12413
CG12481
CG12508
CG12539
CG12746
CG12811
CG12877
CG12951
CG12206
CG12209
CG12229 /// CG14101
CG12236
CG12307
CG12309
CG12325
CG12355
CG12377
CG12378
CG12395
CG12424
CG12432
CG12470
CG12479
CG12493
CG12498
CG12511
CG12521
CG12609
CG12617
CG12637
CG12661
CG12684
CG12689
CG12699
CG12728
CG12730
CG12825
CG12853
CG12857
CG12858
CG12860
CG12861
CG12868
CG1288 /// DbuzCG1288
CG12902
CG12907
CG12945
CG12992
CG13008
CG13075
CG13078
CG13110
CG13168
CG1317
CG13202

CG1299
CG13003
CG13004
CG13014
CG13033
CG13038
CG13040
CG13041
CG13042
CG13043
CG13044
CG13046
CG13047
CG13059
CG13060
CG13063
CG13064
CG13066
CG13067
CG13068
CG13069
CG13101
CG13117
CG13175 /// CG33964
CG13183 /// DsecCG13183
CG13204
CG13220
CG13228
CG13231
CG13319
CG13349
CG13365
CG13392
CG13430 /// CG18065
CG13516
CG1354
CG13545
CG13550
CG13641
CG13654
CG13676
CG13678
CG1368
CG13698
CG13700
CG13722
CG13731
CG1324
CG13243
CG13244
CG13245
CG13323
CG13324 /// DyakGE13403
CG13337
CG13340 /// DmauCG13340 /// DsimGD25742
CG13344
CG13362
CG13385
CG1340
CG13405
CG13422 /// CG7655
CG13424
CG13461
CG1347
CG13473
CG13476
CG13479
CG13488
CG13494
CG13506
CG13511
CG13526
CG13538
CG13539
CG13540
CG13544
CG13564
CG13581
CG13597
CG13602
CG13604
CG13624
CG13693
CG13704
CG13723
CG13733
CG13745
CG13838
CG13841 /// CG4000
CG13843
CG13871
CG13884
CG13898
CG1394

CG13766
CG13795 /// DsecCG13795
CG13796
CG13833
CG13857 /// DmauCG13857 /// DsimCG13857
CG13876
CG13894
CG13902
CG13905
CG13912
CG13917
CG13919
CG13947
CG13962
CG13966
CG14043
CG14062
CG14089
CG14095
CG14118
CG14141
CG14182
CG14184
CG14191 /// DmauCG14191 /// DsimCG14191
CG14195
CG14196
CG14215
CG14222
CG14223
CG14225
CG14244
CG14257
CG14265
CG14270
CG14275
CG14291 /// DpseGA12883 /// DvirGJ14185
CG14299
CG14321
CG14375
CG14397
CG14401
CG1443
CG14435
CG14439
CG14454 /// CG32453
CG14476
CG14482
CG1399
CG14000
CG14022
CG14023
CG14042 /// SP555
CG1407
CG14071
CG14077 /// Ir75d
CG1409
CG14113
CG14154
CG14164 /// CG6709
CG14183
CG14269
CG14294
CG14315
CG14339 /// DsecGM16790
CG14367
CG14383
CG14408
CG14440
CG14442
CG14448
CG14457
CG14471
CG14479
CG14480
CG14488
CG14505
CG14509
CG14556
CG14567
CG14589
CG14608
CG14609 /// DpseGA13108
CG14610
CG1463
CG14631
CG14642
CG14658
CG14659
CG14684
CG14691
CG14695
CG14709
CG14712
CG14718 /// DmauCG14718 /// DsimGD18777

CG14511
CG14512
CG14526
CG14528
CG14561 /// DmojCG14561 /// DvirCG14561
CG14564
CG14570
CG14629
CG14641
CG14642
CG14671
CG14681 /// Skeletor
CG14686
CG14688
CG14710
CG14749
CG14752
CG14810
CG14826
CG14856
CG14881
CG14946
CG14963
CG15021
CG15022
CG15046
CG15116
CG1516
CG15170
CG15201
CG15209
CG15212
CG15282
CG15313
CG15317
CG1532
CG15343 /// DsimCG15343
CG15353
CG15404
CG15406
CG15408
CG15414
CG15422
CG15431
CG15528
CG15531
CG15544
CG14731 /// CG2921
CG14759
CG14763
CG14838
CG14850
CG14852
CG14861
CG14879
CG14898
CG14926
CG14974
CG14995
CG15019
CG15025
CG15034
CG15035
CG15040
CG15043 /// DsimCG15043
CG15065
CG15067
CG15099 /// DmirCG15099
CG15109
CG15125
CG15127
CG15128
CG1513
CG15136
CG15145
CG15177
CG15203
CG15208
CG15219
CG15258
CG15260
CG15286
CG15296
CG15306
CG15314
CG15323 /// DsimCG15323
CG15333
CG15347
CG15387
CG15403
CG15418
CG15423
CG15425
CG15434

CG15695
CG15701
CG15706
CG15727
CG15771
CG15772
CG15773
CG15822
CG15896
CG1607 /// Tpi
CG1632
CG16733
CG16734
CG1675
CG1678
CG16786
CG16790
CG16798
CG16812
CG16820
CG16892
CG16903
CG16926
CG1695
CG16989
CG17002
CG17026
CG1707
CG17124
CG17184
CG17190
CG17192
CG17224
CG17249
CG17259
CG17264
CG17282
CG1732
CG17322
CG17323
CG17327
CG9133
CG17331
CG17333
CG17360
CG17556 /// CG3678
CG17571
CG15452
CG15461
CG15465
CG15475
CG15482
CG15510
CG15530
CG15533
CG15543
CG15576
CG15580
CG15605
CG15625
CG15631
CG15657
CG15676
CG15705
CG1571
CG15711
CG15719
CG15734
CG15745
CG15765
CG15800
CG15818 /// DsimCG15818
CG15870
CG15873
CG15878
CG15909
CG15922
CG1625
CG1631
CG1657
CG16711
CG16716
CG1673
CG16736
CG16739
CG16741
CG16758
CG16762
CG16782
CG1681
CG16836
CG16848
CG16854
CG16888

CG17597 /// ScpX
CG17662
CG17680
CG17712
CG17721
CG17726
CG1773
CG17751
CG17754
CG17760
CG17776
CG17840
CG17919
CG1792
CG17928
CG18004
CG1809
CG18095
CG18249
CG18259
CG18343
CG18446
CG18493
CG18542
CG32939
CG18549
CG18557
CG18577
CG18585
CG18600
CG1887
CG1896
CG1902
CG1927
CG1942
CG1968
CG1969
CG2023
CG2034
CG2065
CG2076
CG2091
CG2144
CG2147
CG2177
CG2199
CG2211
CG16894
CG16941
CG16964
CG16972
CG16995
CG17003
CG17010
CG17019
CG17030
CG17048
CG17083
CG17108 /// CG40263
CG17109
CG17141
CG17154
CG17196
CG17217
CG17237
CG1724
CG17258
CG1732 /// CG6218
CG17344
CG17350
CG17376
CG17377 /// os
CG17380
CG17387
CG17470
CG17567
CG17598
CG17612
CG17618
CG6982
CG17625
CG17648
CG17666
CG17669
CG17724 /// seq
CG17744
CG17764
CG17856
CG17904
CG17930
CG17944
CG18067
CG18107
CG18109

CG2277
CG2493 /// DsecGM23324
CG2543
CG2604 /// DsimGD19638
CG2641
CG2663
CG2680
CG2767
CG2774
CG2781
CG2812
CG2818
CG2889
CG2909
CG2930
CG2941 /// CG32783 /// CG32786
CG2943
CG2962
CG30016
CG30031 /// CG4269
CG30091
CG30100 /// CG42372
CG30157
CG30172
CG30196
CG30197
CG30217
CG3033
CG30359
CG30360
CG30377
CG30380
CG30381
CG30392
CG30410
CG30423
CG30427
CG30431
CG30466
CG30503 /// kappaB-Ras
CG31030
CG31075 /// DsecCG31075
CG31100
CG31104
CG31111
CG31125
CG31145
CG18132
CG18171
CG18179
CG18193
CG1826
CG18273
CG1832
CG1835
CG18371
CG18404
CG18410
CG18418
CG18449
CG18536
CG18539
CG18545
CG18598
CG18619
CG18635
CG18649
CG18662
CG18666
CG18675 /// tipE
CG18744
CG18749 /// CG33722
CG18810
CG18853 /// phr
CG1958
CG1979
CG1988
CG1999
CG2006
CG2053
CG2083
CG2137
CG2225
CG2249
CG2577
CG2616
CG2750
CG2854
CG2861
CG2865
CG2871
CG2926
CG30008
CG30022 /// CR40100

CG3117
CG31223
CG31272
CG31274 /// MESK4
CG31321
CG31344
CG31370
CG31373
CG31406
CG31446
CG31460
CG31496
CG31522
CG31548
CG31549
CG31663
CG31672
CG3168
CG31683
CG31687 /// CG31688
CG31698
CG31729
CG31775 /// CG42586
CG31778
CG31809
CG31810
CG3184
CG31917 /// Tfb5
CG31919 /// CG33995
CG3192
CG31937
CG31953 /// gkt
CG31955
CG32023
CG32039
CG32069
CG32073
CG32115
CG32187
CG32191
CG32195
CG32198
CG32212
CG32267
CG32281
CG32425
CG32441
CG30026
CG30039
CG30056
CG30058 /// CG34235 /// CG34312
CG30099
CG30108 /// CG30109
CG30110
CG30178
CG30192
CG30194
CG30222
CG30271
CG30278
CG30281
CG30286
CG30321
CG30324
CG30334
CG30343
CG30354
CG30355
CG30356
CG30369
CG30384
CG33140
CG30389
CG30393
CG30398
CG30412
CG30432
CG30441
CG30459
CG30460
CG30461 /// ste24c
CG30487
CG30497
CG30502
CG3056
CG3062
CG3085
CG31004
CG31007
CG31008
CG31029
CG31055
CG31087
CG31091

CG32447
CG32495 /// GS
CG32500 /// CG32857 /// CG33502
CG32506
CG32536 /// CG32537
CG32579
CG32581 /// CG8974
CG32590
CG32603
CG32626
CG3264
CG32649
CG32662
CG32669
CG32698
CG3277
CG32803
CG3281
CG3285
CG3292
CG33013
CG3303 /// CR31292
CG33051
CG33129
CG33138 /// DsecCG33138
CG33143
CG33145
CG33155 /// mRpL53
CG33268 /// CG7377
CG33272
CG33281
CG3332
CG33346
CG3337
CG3342
CG3344
CG33465
CG3347
CG3348
CG33493
CG33514
CG3355
CG3363
CG33635
CG3376
CG33785 /// CG33786
CG33932 /// Rpp20
CG31161
CG31169
CG31178 /// DsimGD20903
CG31195
CG31206
CG3121
CG31210
CG31213
CG31226
CG31230
CG31231
CG3124
CG31244
CG31286
CG31287 /// Eno
CG31294
CG31323
CG31327
CG31352
CG31407
CG31468 /// DsimGD18362
CG31482
CG31528
CG31531
CG31534
CG31538
CG31542
CG31546
CG31624 /// DsimGD21644
CG31635
CG31639 /// Uch-L3
CG31642
CG31644
CG31679
CG31693
CG31697
CG31702 /// CG31703
CG31709
CG31712
CG31740
CG31742
CG31752
CG31769
CG31773
CG31781
CG31782
CG31788

CG3394
CG33966
CG3397
CG33970
CG34057
CG3407
CG3408
CG34116
CG34125
CG3420
CG34247
CG34248
CG34253
CG34278
CG34279
CG34331
CG34349
CG34350
CG3436
CG34386
CG34396
CG34408
CG34422
CG34445 /// CG34446
CG3446
CG3500
CG3502
CG3505
CG3511
CG3527
CG3532
CG3556
CG3560
CG3570
CG3588
CG3589
CG3603
CG3618
CG3621
CG3632
CG3652
CG3663
CG3690
CG3706
CG3731
CG3734
CG3735
CG31802
CG31804
CG31806
CG31815
CG31816
CG31861
CG31870
CG31874
CG31894
CG31907
CG31909
CG31910
CG31920
CG31924
CG31958
CG31960
CG31974
CG31988
CG3199
CG32000
CG32026
CG32043
CG32063
CG32064 /// DsimGD14244
CG32117
CG3213
CG32138
CG32141
CG32148
CG32152
CG32163
CG32181
CG3222
CG32240
CG32284
CG32295
CG32298 /// CG32299
CG32313
CG32368
CG32371
CG32379
CG32388
CG32396
CG32437
CG32440
CG32450
CG32459

CG3797
CG3803
CG3819
CG3823
CG3829
CG3842
CG3857
CG3894
CG3984
CG40002
CG4019
CG4020
CG4041
CG4045
CG4080
CG4096
CG4168
CG4169
CG4210
CG42231 /// chif
CG42235
CG42246
CG42255
CG42269
CG42327
CG42336
CG42370
CG4250
CG42511 /// CG8135
CG42542
CG42575
CG4259
CG4278
CG4287
CG4288
CG4293
CG4302 /// DsecCG4302
CG4306
CG4330
CG4332
CG4338
CG4365
CG4382
CG4386
CG4390 /// DpseGA18152
CG4398
CG4400
CG32461
CG32541 /// CG42506
CG32568
CG32573 /// CG42512
CG32582
CG32588
CG32628
CG32655
CG32663
CG32686
CG32694
CG32699
CG32700 /// DsimGD16059
CG32703
CG32713 /// CG33223 /// DsimCG32713
CG32758
CG32791
CG32806
CG32809
CG32829
CG32832
CG32835
CG32845
CG32945
CG32971
CG32982
CG33054
CG33057
CG3306
CG33111
CG33136
CG33189
CG33199 /// CG8229
CG33218
CG33225
CG33229
CG3323
CG33232
CG33235
CG33284
CG33293
CG3330 /// DwilGK11338
CG33308
CG33322
CG33340
CG3345
CG33462

CG4406
CG4407
CG4408
CG4409
CG4447
CG4462
CG4496
CG4500 /// DsecCG4500
CG4502
CG4572 /// DpseGA18267
CG4594
CG4627
CG4630
CG4645
CG4646
CG4662
CG4670
CG4702
CG4721
CG4725
CG4743
CG4749
CG4752
CG4774
CG4785
CG4789
CG4797
CG4822
CG4842
CG4860
CG4866
CG4884
CG4908
CG4927
CG4945
CG4950
CG4953
CG4973 /// DpseGA18564
CG4975 /// CG4984
CG5002
CG5013
CG5021
CG5033
CG5112
CG5151
CG5162
CG5167
CG33469
CG33470
CG33489
CG33541
CG33691 /// CG33692
CG33773
CG33937
CG33960
CG33969 /// fbl
CG33978
CG34005
CG34021
CG34026
CG34029
CG3409
CG34104 /// Tequila
CG34110
CG34114
CG34126
CG34161
CG34168
CG34171
CG34175
CG34188
CG34204
CG34210
CG34230
CG34231
CG34245
CG34261
CG34265
CG34269
CG34274
CG34283
CG34300
CG34347
CG34354
CG34360
CG34386
CG34394
CG34398
CG34400
CG34401
CG34409
CG34417
CG34449
CG3492

CG5174
CG5180
CG5191
CG5210
CG5224
CG5254
CG5292
CG5315
CG5321
CG5322
CG5325
CG5339
CG5362
CG5412
CG5414
CG5439
CG5466
CG5484
CG5532
CG5535
CG5577
CG5612
CG5618
CG5630
CG5641
CG5656
CG5703
CG5756
CG5758
CG5770
CG5773
CG5830
CG5844
CG5854 /// DgriCG5854 /// DmojCG5854 /// Dvir[illegible]
CG5861
CG5867
CG5885
CG5946 /// zetaCOP
CG5976
CG5989
CG5999
CG6020
CG6067
CG6084
CG6126
CG6195
CG6196
CG3517
CG3565
CG3581
CG3700 /// DmirCG3700 /// MED23
CG3719
CG3732
CG3740
CG3777
CG3800
CG3809
CG3814 /// TTLL3B
CG3875
CG3884
CG3940
CG3982
CG40115
CG40178 /// CG41320
CG40470
CG40625 /// JYalpha
CG41284 /// CG42598
CG4194
CG42232
CG42258
CG42288
CG42330
CG42335
CG42355
CG42374 /// CG9666
CG42383
CG42388
CG42390
CG42394
CG42448
CG42450
CG42457 /// cpo
CG42487
CG42493
CG42533
CG42559 /// CG42560
CG42565
CG42568 /// DnaJ-60
CG42570
CG42574
CG42579 /// CG42580
CG42588
CG42591
CG42593

CG6204
CG6231
CG6276
CG6283
CG6293
CG6294
CG6299
CG6295
CG6296
CG6340
CG6356
CG6361
CG6370
CG6388
CG6410
CG6459
CG6506
CG6511
CG6540
CG6583
CG6607
CG6613
CG6617
CG6621
CG6650
CG6656
CG6660
CG6704
CG6726
CG6729
CG6746
CG6753
CG6758 /// DmirCG6758
CG6805
CG6834
CG6910
CG6915
CG6928
CG7011
CG7016
CG7083
CG7084
CG7115
CG7133
CG7145
CG7146
CG7172
CG42613
CG42637
CG4266
CG4270
CG4297
CG4329
CG4362
CG4367
CG4375
CG4477
CG4546
CG4562
CG4619
CG4623
CG4631
CG4661
CG4662
CG4680
CG4681
CG4683
CG4706
CG4716 /// DsimGD10957
CG4744
CG4757
CG4810
CG4825 /// DsecGM22203
CG4829
CG4836 /// DsecGM17688
CG4839
CG4857
CG4911
CG4955
CG4956
CG4962 /// DmauCG4962 /// DsimCG4962
CG4988
CG5011
CG5017
CG5024
CG5048
CG5062
CG5071
CG5089
CG5116
CG5122
CG5139
CG5144
CG5204

CG7179
CG7181
CG7191
CG7203
CG7215 /// Prx5
CG7246
CG7275
CG7277
CG7290
CG7294
CG7300
CG7322
CG7330
CG7342
CG7367
CG7372
CG7402
CG7430 /// DsimCG7430
CG7442
CG7448
CG7466
CG7470
CG7497
CG7518
CG7519
CG7536
CG7550
CG7567
CG7582
CG7587
CG7603
CG7607
CG7627
CG7630
CG7671
CG7675
CG7685
CG7695
CG7713
CG7745
CG7763
CG7772
CG7777
CG7789
CG7824
CG7830
CG7840
CG5217
CG5265
CG5280
CG5326
CG5357
CG5398
CG5399
CG5435
CG5538
CG5550
CG5556
CG5565 /// DsimCG5565
CG5614
CG5664
CG5762 /// DmauCG5762 /// DsimGD18296
CG5778
CG5781
CG5840
CG5853
CG5872
CG5953
CG5968
CG5987
CG6006
CG6045
CG6049
CG6083
CG6138
CG6140
CG6175
CG6182
CG6209
CG6271
CG6279
CG6327
CG6330
CG6337
CG6340
CG6357
CG6372
CG6380
CG6441
CG6497
CG6527
CG6569
CG6629
CG6738

CG7860
CG7882
CG7906
CG7914
CG7916
CG7920
CG7943
CG7949
CG7997
CG8012
CG8021
CG8031
CG8067
CG8083
CG8100
CG8111
CG8112
CG8132
CG8159
CG8180
CG8193
CG8211
CG8229
CG8232 /// Rcp
CG8239
CG8270
CG8306
CG8314
CG8315
CG8326 /// DyakGE15584
CG8360
CG8372
CG8389
CG8412
CG8443
CG8446
CG8507
CG8519
CG8543
CG8550
CG8560 /// DsecCG8560
CG8562
CG8586
CG8661
CG8668
CG8768
CG8778 /// DmirCG8778
CG6745
CG6751
CG6763
CG6789
CG6873
CG6888
CG6893
CG6901
CG6905
CG6967
CG6971
CG6980
CG6996
CG7024
CG7029
CG7039
CG7045
CG7046
CG7059
CG7069
CG7120
CG7164
CG7182
CG7188
CG7202
CG7211
CG7229
CG7248
CG7262
CG7264
CG7276
CG7295
CG7324
CG7337
CG7357
CG7366
CG7422
CG7431
CG7441 /// DsimGD14730
CG7514
CG7557
CG7638
CG7650
CG7669
CG7707
CG7768
CG7813

CG8786
CG8889
CG8928
CG8931
CG8965
CG8997 /// DsimCG8997
CG9018
CG9034
CG9065
CG9067
CG9090
CG9107
CG9119
CG9125
CG9154
CG9172 /// TSG101
CG9186
CG9205
CG9220
CG9257
CG9259
CG9267
CG9302
CG9306
CG9312
CG9331
CG9368
CG9372
CG9394
CG9427
CG9452
CG9458
CG9486
CG9505
CG9572
CG9576
CG9577
CG9616
CG9662
CG9664
CG9682
CG9689
CG9702
CG9717
CG9769
CG9801
CG9812
CG7839
CG7886
CG7907
CG8008
CG8038
CG8060
CG8066
CG8078
CG8086
CG8087
CG8097
CG8129
CG8187
CG8206
CG8292
CG8297
CG8299
CG8320
CG8405
CG8407
CG8494
CG8500
CG8517
CG8526
CG8557
CG8564
CG8620
CG8630
CG8671
CG8701
CG8712
CG8746
CG8750
CG8840
CG8851
CG8852
CG8907
CG8944
CG8949
CG9007
CG9016
CG9062
CG9086
CG9106
CG9129
CG9130 /// CG9133
CG9194

CG9813
CG9826
CG9837
CG9867
CG9870
CG9903
CG9914 /// DsecCG9914
CG9917
CG9934
CG9945
CG9953
CG9987
CG9989
CG9996
Chrac-16
Cht11
Cht2
ck
Cka
ckd
ClC-b
cln3
clt
cni
cnir
Cog7
colt
Cp18
Cp38
Cpr30F
Cpr64Ab
Cpr64Ad
Cpr65Av
Cpr65Ea
Cpr65Ec
Cpr66Cb
Cpr67Fa1 /// Cpr67Fa2
Cpr78Ca
Cpr97Ea
CR32207
Cralbp
crim
croc
CSN6
CstF-50
csul
CtBP
CG9254
CG9263 /// Yp1
CG9281
CG9314
CG9328
CG9389
CG9445
CG9498
CG9550
CG9570
CG9624
CG9650
CG9733
CG9836
CG9875
CG9894
CG9896
CG9902
CG9920
CG9948
CG9975
CHES-1-like
chinmo
CHMP2B
chn
chrb
chrw
Cht12
Cht4
cic
cindr
Cip4
CkIalpha
CLIP-190
cmet
cno
cora
cp309
Cpr47Eb
Cpr51A
cpx /// SNF4Agamma
CR18854
CR32690
crol
crp
CS-2
CTCF

Ctr1A
cyc
Cyp12c1
Cyp12d1-d
Cyp12d1-p
Cyp12e1
Cyp304a1
Cyp309a1
Cyp4ad1
Cyp4d1
Cyp4d2
Cyp4e2
Cyp4p1
Cyp6a17
Cyp6d2
Cyp6d5
Cyp6g1
Cyp6g2
Cyp6t1
Cyp6v1
Cyp6w1
dan
danr
daw
dbr
Dcp1
Der-1
dik
Dim1
Dip-B
DNApol-alpha73
DNaseII
dpn
Drip
dro4
dro5
dsx
E(spl)
E(var)3-9
e(y)1
ea
Eaat1
eater
ECSIT
Ect3
Edem1
edl
Ctr1B
cv-c
CycT
Cyp28a5
Cyp310a1
Cyp4g1
Cyp4p2
Cyp4p3
Cyp6a18
Cyp6a23
Cys
Cyt-c-d
Damm
Dcp2
Def
Dgk
Dhap-at
Dhc36C
dia
Dif
disco
djl
dl
dlg1
dlp
dm
Doa
DopEcR
dpld /// wech
DptB
Dpy-30L2
Drak
Drep-2
drk
drm
dro2
dro3
Drs
Dscam
dsx
dtr
dve
Dys
E(bx)
E(Pc)
ed
Edc3

EDTP
Efr
egg
eIF2B-beta
eIF2B-delta
eIF4AIII
Eig71Ee
Eip75B
elB
elgi
Elp2
ema
Eo
epsilonCOP
epsilonTry
erm
esg
ey
Fas2
fat-spondin
fau
fd96Ca
Fdh
ferrochelatase
Fhos
flr
fok
for
fra
fray
frj
fu
Fur1
fusl
fy
Gal
Gapdh1
Gapdh2
Gas41
gb /// Oatp58Db
gcm
gek
Gen
Gfr
Gip
GlcAT-I
GlcAT-P
Edg91
eIF-4B
eIF4E-4
eIF4E-7
eIF4G2
Eip75B
elav
endoB
ERR
ex
exex
exu
faf
fan
fau
fd68A
Fie
Fili
Fim
Fip1
Fis1
fkh
FLASH
foxo
fru
fry
fs(1)h
Fs(2)Ket
ftz-f1
fzo
G9a
Galpha49B
Gap1
garz
GATAd
gdl /// gdl-ORF39
Gem3
Gfat1
Gie
gish
glec
glob2
Glycogenin
gom
grp
Grx-1
GstD2

GlcT-1
Gli
GlyP
Gmer
GNBP3
Gpdh
Gpi1
granny-smith
Grasp65
grass
GRHR
Gs1
Gs1l
Gs2
GstD3
GstE10
GstE3
GstE7
GstE9
GV1
H15
HBS1
HDAC6
He
Hem
Hexo2
Hf
His3.3A
HLHm7
HLHmdelta
Hml
hoe2
HP1b
HP1c
HPS1
Hr39
Hr78
Hrd3
Hs2st
Hsp22 /// Hsp67Bb
Hsp67Bc
ihog
Ilp2
Ilp3
ImpL2
inaF-D
Indy
Gug
hep
her
Herp
Hex-A
hgo
how
HP1e
HP6
hrg
Hsc70-1
Hsc70-2
Hsp22 /// Hsp67Bb
Hsp60B
Hsp68
Hsp70Aa /// Hsp70Ab
Hsp70Ba /// Hsp70Bb /// Hsp70Bbb /// Hsp70Bc
htt
ia2
ifc
ik2
IM1
IM10
IM2
IM23
IM3
Imp
inaE
insc
IP3K1
I-t
itp
janB
jim
jing
Jupiter
kay
kek5
kel
klg
Klp10A
Klp59C
Klp98A
kst
Kua
kuz
l(1)G0045

Ing3
Ino80
Invadolysin
iPLA2-VIA
Ipp
isopeptidase-T-3
jdp
Jhe
Jhedup
Jheh2
Jon25Biii
Jon65Aii
Jon66Cii
Jon74E
Jon99Ci
Jon99Fi
jub
kar
Kaz1-ORFA /// Kaz1-ORFB
kek1
KLHL18
Klp64D
kn
knk
Kr
l(1)G0004
l(1)G0007
l(1)G0144
l(1)G0156
l(1)G0230
l(2)01289
l(2)01810
l(2)03659
l(2)35Di
l(2)37Cb
l(2)efl
l(2)k09913
l(2)not
LanA
Las
Lcch3
Lcp2
Lcp65Ac
lectin-24Db
lectin-28C /// ninaE
Leucokinin
lig3
l(1)G0196
l(1)G0469
l(2)k16918
l(3)neo38
Lasp
ldlCp
lectin-37Da /// lectin-37Db
lectin-46Ca
Lerp
lilli
Lim3
Listericin
lola
LRP1
LysX
Madm
mahj
mam
Mapmodulin
mask
Mef2
mei-217 /// mei-218
mei-P26
Menl-1 /// Menl-2
MESK2
mew
Mical
milt
mirr
Mkp3
Mmp1
mnb
Mnt
mod(mdg4)
mof
Mrtf
msl-3
msn
mspo
msps
Mst35Ba
Mst35Bb
Mst77F
Mst84Da
Mst84Db
Mst84Dc
Mst84Dd

lin19
LKR
loj
Lsp1gamma
luna
lva
LvpH
LysP
m4
M6
Mad
MAGE
malpha
Map60
mbm
Mcm2
mdy
MED11
MED14
MED18
MED28
mei-41
Mes2
MESK2
MESR6
Mf
Mfap1
mfas
Mgstl
Mip
miple
mira
mirr
Mkrn1
mnd
mod(mdg4)
mor
MRG15
mrn
mRpL10
mRpL17
mRpL18
mRpL23
mRpL28
mRpL30
mRpL41
mRpL48
Mst87F
Mst89B
Mst98Cb
mthl1
mthl10
Mtk
Mtl
MtnB
Mtor
mts
mtsh
Muc18B
Muc26B
Muc55B
Muc68Ca
mud
Mur29B
mura
mus309
Myo95E
myoglianin
MYPT-75D
mys
NaCP60E
nAcRbeta-64B
Ndae1
Nedd4
Nedd8
Nep3
NFAT
nimC3
nimC4
nito
nkd
nol
nompB
Nop60B
nopo
norpA
Not1
Notum
Nrg
ns2
n-syb
Ntl
nub
nuf

mRpS11
mRpS18B
mRpS18C
mRpS2
mRpS22
mRpS28
mRpS31
mthl3
mthl4
mthl5
mthl6
mthl8
mtm
Mur18B
Mur89F
Myo28B1
Myt1
nAcRbeta-21C
Nc
ND23
Nelf-E
neo
Nep1
Nep2
net
NetB
nimB1
nimB5
nimC1
nimC2
NitFhit
NLaz
Nmdmc
Nmt
noc
Nop56
Npc2c
Npc2d
Npc2g
Nplp4
Nrg
Nrk
nrv1
NtR
Nurf-38
oaf
Oatp58Da
Nup160
nxf4
Oatp33Eb
Obp50e
obst-H
obst-J
oc
okr
olf186-F
omd
ome
os
Pabp2
Paip2
Pak
pan
par-1
para
Pbp95
pcl
Pde1c
Pde6
pdm2
Pepck
PGRP-LF
PGRP-SB1
phr
Pif1A
pigs
Pino
Pka-C1
Pka-R2
Pkd2
PMCA
Pnn
pnt
PNUTS
Pof
por
Porin2
PP2A-B
PpD5
PpY-55A
PPYR1
PQBP-1
pr
pros

Obp56a
Obp56e
obst-A
obst-B
Odc1
O-fut1
O-fut2
Opbp
Orc6
Orct
ORMDL
Osi21
Osi7
Ost48
p120ctn
p24-2
pall
Papss
Pbgs
PCID2
pea
PebIII
PEK
Peritrophin-A
pex1
pex12
pex13
Pfk
Pgam5
Pgant35A
pgant8
Pgd
Pgk
Pgm
PGRP-SA
PGRP-SC1a /// PGRP-SC1b
PH4alphaEFB
phm
Pi3K21B
Pi4KIIalpha
Pink1
pio
Pis
pnr
Pole2
POSH
Ppt1
Prosbeta2R2
Psf3
psq
Ptp10D
Ptp4E
Ptp61F
Ptp99A
puc
pum
pyd
pygo
qjt
qua
qvr
Rab2
rab3-GEF
Rab6
Rala
ran-like
Rbp2
Rcd2
rdx
rec
Rfx
rha
RhoGAP1A
RhoGAP71E
rig
Rip11
rk
rl
r-l
rn
RN-tre
robl22E
robl62A
Roc1b
rok
roq
row
Rox8
Rpb10
RpL10Aa
RpL12
RpL3
RpS28-like
RpS7

Prat2
Prestin
proPO-A1
Pros28.1
Prp18
Prx6005
Psc
Psf1
psh
Psn
Ptpa
Ptr
Pxd
Pxn
pyd3
PyK
Rab35
Rab4
Rae1
raptor
Rbcn-3A
Rbp1
Rca1
rdgC
regucalcin
repo
rept
rev7
RfC38
RFeSP
Rh6
rho-5
ric8a
Rlb1
RnpS1
rod
rost
RpII140
RpL13A
RpL28
RpL34b
Rpn1
Rpn12
Rpn3
RpS11
Rpt3
Rrp4
rut
RYBP
S
SAK
sala
Sap47
sas
sbb
sbr
scb
scramb1
s-cup
scyl
sda
Sdic1
Sdic3 /// Sdic4 /// sw
Sema-1a
seq
Ser
Ser6
Sh
shakB
Shal
shep
shi
Shroom
Sin3A
SIP2
skd
skpD
slik
sm
Smg6
sno
sns
Socs36E
sog
Sox100B
Sox15
Spargel
spaw
spel1
spheroide
spir
Spn
Spn47C
spri

Rrp46
rtet
rtv
rumi
S6kII
sad
Saf-B
salt
SamDC
santa-maria
sbb
Scamp
Sce
sced
scu
SdhB
SdhC
Sdic3
sec15
Sec16
sec5
sec6
SelR
Sfmbt
Sgs1
Sgs5
shark
Side
SIDL
sinah
Sirt4
Slh
slif
slmb
SmD3
Snr1
Sodh-1
Sox14
Sp1
Sp7
Spase25
Spc25
spdo
SPE
spict
Sply
Spn1
Src64B
SRm160
Srp54k
ssh
ssp2
ssp4
ssp5
Stim
stwl
sty
Su(dx)
Su(var)3-7
sun
sunz
sut4
swi2
Syb
Syt1
Taf13
Taf4
tai
Tango13
Tao-1
t-cup
Tdc1
Tektin-C
Tequila
Thd1
thoc6
Tim13
Tim17a1
Tim17b1
Tim17b2
timeout
tio
Tk
tlk
Tm1
tmod
tomboy20
Top1
Top3beta
TORC
TotA
toy
Tpl94D
Traf-like

Spn100A
Spn28D
Spn43Aa
Spn43Ab
Spn88Eb
spn-E
Spred
Spt3
Spt5
Spt-I
Sptr
spz3
spz6
Sr-CI
Srp68
SsRbeta
Stam
ste14
sty
su(f)
Su(fu)
Sucb
sug
Surf1
sxc
Tace
Taf10
Taf7
Tak1
Takl1
Tal
Tango5
Tango6
Tango9
tap
Tap42
Tapdelta
Tcp-1eta
tef
Teh3
TepI
TepII
tex
TFAM
Tfb4
tgo
thetaTry
Trf4-1
TrxT
Tsp42Ec
Tsp66A
Tsp96F
tst
ttm3
tty
tup
tutl
tutl
Uba1
Ubc84D
Ucp4B
UGP
Ugt86Dj
unk
Unr
VAChT
vav
vg
Vha14-2
Vha16-1
Vha16-2
Vha36-2
Vha68-3
VhaM9.7-d
vir-1
Vmat
vn
Vsx1
wapl
w-cup
wda
wdb
wkd
XRCC1
yellow-d2
ymp
yuri
Zasp66
zfh1
zfh2
zormin

Thiolase
thoc5
tko
Tm1
TM4SF
Top3beta
tra2
Trc8
trus
Tsf1
Tsf2
Tsp3A
Tsp42Ej
Tsp42Ek
Tsp42Ep
Tsp74F
Tsp86D
TwdlC
TwdlH
TwdlK
TwdlT
U2A
Ubc-E2H
Ugt36Bc
Ugt37b1
UK114
Unc-89
up
usnp
VhaM9.7-a
VhaM9.7-c
vkg
vls
Vmat
vtd
w
wbl
wg
wibg
wor
WRNexo
Wsck
wuho
wun2
wus
yellow-b
yellow-d

yellow-e3
yellow-f
yemalpha
yin
Yp3
zetaCOP
zormin
Zw
zwilch
